# Non-human papillomaviruses for gene delivery in vitro and in vivo

**DOI:** 10.1371/journal.pone.0198996

**Published:** 2018-06-18

**Authors:** Lea Bayer, Jessica Gümpel, Gerd Hause, Martin Müller, Thomas Grunwald

**Affiliations:** 1 Department of Immunology, Fraunhofer-Institute for Cell Therapy and Immunology IZI, Leipzig, Germany; 2 Biozentrum, Martin-Luther-University Halle-Wittenberg, Halle (Saale), Germany; 3 German Cancer Research Center, Heidelberg, Germany; Albert Einstein College of Medicine, UNITED STATES

## Abstract

Papillomavirus capsids are known to have the ability to package DNA plasmids and deliver them both *in vitro* and *in vivo*. Of all known papillomavirus types, human papillomaviruses (HPVs) are by far the most intensely studied. Although HPVs work well as gene transfer vectors, their use is limited as most individuals are exposed to this virus either through a HPV vaccination or natural infection. To circumvent these constraints, we produced pseudovirions (PsVs) of ten non-human papillomavirus types and tested their transduction efficiencies *in vitro*. PsVs based on Macaca fascicularis papillomavirus-11 and Puma concolor papillomavirus-1 were further tested *in vivo*. Intramuscular transduction by PsVs led to months-long expression of a reporter plasmid, indicating that PsVs have potential as gene delivery vectors.

## Introduction

The transfer of nucleic acids for gene therapy or as genetic vaccines has several advantages, most importantly the rapid production, simple adaptation and high stability at ambient temperature of DNA. Additionally, the recipient’s cells themselves express the encoded antigens, allowing correct post-translational modifications and folding of the protein. Immunization with a DNA vaccine activates both the humoral and cellular immune response, making genetic vaccines a powerful platform [1]. While intramuscular injection of naked DNA leads to a reasonable cellular uptake and subsequent expression in rodents [2], larger animals—especially non-human primates—require additional stimuli to enhance the uptake of the plasmid DNA [3]. One of the most effective methods to enhance DNA-uptake is the use of electroporation [4,5]. However, electroporation is an invasive and painful procedure, requiring local anesthesia and the presence of special equipment [6]. Other delivery methods include physical devices such as pressure injector, gene gun [7], and chemical formulations such as block copolymers [8], cationic liposome [9] and polyethyleneimine [10] and calcium nanoparticles [11]. Furthermore, the application of a variety of bacteria and viruses as gene carriers has been explored, human papillomaviruses being one of them [12]. Several characteristics of papillomaviruses make them promising candidates as DNA delivery vectors, such as their stability due to being non-enveloped, their ability to package foreign DNA up to 8kbp of without the need of a specific packaging sequence [13] and their capability to infect mucosal tissue [14]. Gene delivery using papillomaviruses however does face the same issues as other more commonly used viruses: the problem of existing immunity against the vector.

Human papillomaviruses (HPVs), especially type 16, work quite well as gene delivery vehicles [12,15], however, more and more virus like particles (VLPs) of different papilloma types are being added to vaccines against HPV. Merck’s “Gardasil 9”, approved by the FDA in December 2014, includes VLPs of HPV types 6, 11, 16, 18, 31, 33, 45, 52, and 58 [16]. It would consequently not be possible to apply these papillomavirus types as gene carriers in *Gardasil 9* vaccinated individuals. In addition to vaccine-induced immunity against HPV, natural infection occurs quite frequently, making it difficult to reliably apply HPV for the delivery of genetic vaccines in the general population.

In 2013, 260 different papillomavirus types were identified, among those 148 human and 112 non-human-papillomavirus (NHPV) types [17]. We therefore set out to explore the *in vitro* production of non-human papilloma pseudovirions (PsVs) and their ability to deliver DNA-plasmids *in vitro* and *in vivo*. Published sequences for capsid proteins L1 and L2 of ten NHPVs were codon-optimized for expression in human cell lines, synthesized and cloned into expression vectors. We produced VLPs and PsVs following the protocol established by Buck et. al with slight modifications [18].

We find that one of the ten NHPVs tested shows high transduction efficiencies both *in vitro* and *in vivo*, and might be suitable for further evaluation as a vaccine platform or as a vector in a gene therapy setting.

## Materials and methods

### Cells

HEK293TT (kindly provided by CB Buck, National Cancer Institute, USA) cells were maintained in Dulbecco’s modified Eagle medium (DMEM), supplemented with 10% fetal bovine serum (Gibco), 100U/ml Penicillin-Streptomycin (Gibco), GlutaMAX and 25mM glucose. The 293TT cell line was originally generated by transfection of 293T cells with linearized pTIH plasmid to achieve an increased expression of the SV40 Large T antigen [13]. For selection, 400μg/ml of hygromycin (Santa Cruz) were added to cell culture medium.

### Plasmid construction and propagation

DNA-sequences coding for papillomavirus capsid proteins L1 and L2 were codon-optimized for expression in human cells using GeneArt’s GeneOptimizer and synthesized by GeneArt (Invitrogen, Regensburg, Germany). Sequences are available in the supporting information S1 File. L1 and L2 sequences were cloned into the multiple cloning site of the mammalian expression vector pcDNA3.1+ (Invitrogen) via HindIII and XhoI. The two sequences were connected by an IRES-sequence (GenBank: X68257.1), which was synthesized by GeneArt and cloned into the vector via AflIII and NheI. Cloning as well as plasmid propagation was performed in *E*.*coli* DH5α (New England Biolabs).

Plasmids for transfection were prepared using a NucleoBond Plasmid PC500 Maxiprep Kit (Macherey Nagel).

As reporter plasmids, pCMV-Gluc-1 luciferase of *Gaussia princeps* (Targeting Systems/NEB)), pCR-Luc3 (firefly luciferase reporter) and pEGFP-C1 (GFP reporter; GenBank U55763.1) were used.

### Transfection

For VLP- and PsV-production, approx. 18h prior to transfection 6x10^6^ HEK293TT cells were seeded in a 75cm^2^ cell culture flask. The transfection mix was prepared by adding 19μg of the plasmid coding for papillomavirus L1 and L2 and 19μg of the reporter plasmid (pEGFP or pCMV-G.Luc) to 1ml of DMEM. Last, 50μl of polyethyleneimine (1mg/ml) were added and the preparation was incubated at room temperature for 10min. Cell culture media was changed to DMEM supplemented with 1.5% FBS and penicillin/streptomycin before adding the transfection mix. Approx. 16h after transfection, media was removed, cells were washed with PBS and complete cell culture media was added.

### VLP and PsV harvest

Harvest of VLPs and PsVs was performed approx. 40 hours after transfection following the standard protocol of Buck et. al [18] with a few modifications. Briefly, cells lysed by adding Triton-X 100 at a final concentration of 0.5% in PBS containing an additional 9.5mM MgCl_2_, 10mM HEPES and 25mM ammonium sulfate. DNA was digested for 24 hours through the addition of Benzonase nuclease (final concentration ≥ 0.25U/μl) and Plasmid Safe exonuclease (final concentration 0.01U/μl). The lysate was chilled on ice and centrifuged at 5,000g for 10min at 4°C. After removal of the VLP or PsV containing supernatant, the remaining cell pellet was resuspended in 100μl of PBS containing an additional 0.8M NaCl. After initial screening experiments, PBS without additional NaCl was used for PcPV1 preparations. The resuspended cells were again centrifuged as above, and supernatant was pooled with the supernatant from the first centrifugation step. This resulting clarified lysate was either stored at -80°C or purified by ultracentrifugation.

Density gradient ultracentrifugation was performed using OptiPrep (Axis-Shield) diluted with PBS + 0.8M NaCl to 27%, 33%, and 39%. Gradients were cast by underlayering in a 5ml tube (Beckman Coulter). Clarified lysate was layered on top, and tubes were centrifuged in an SW 55 Ti rotor (Beckman Coulter) at 50,000rpm (234,000g) for 3.5 hours at 16°C. After centrifugation, the upper layer up to the transition from 27% to 33% OptiPrep was discarded, and subsequently 12 fractions of 250μl each were collected by pipetting into siliconized 1.5ml tubes.

Instead of performing ultracentrifugation with OptiPrep, Percoll (GE Healthcare) was used for purification of MfPV11 and HPV16 PsVs. Clarified supernatant was layered on top of 4.5ml of Percoll diluted to 58.3% with PBS + 0.8M NaCl. Tubes were centrifuged in an SW 55 Ti rotor at 30,000rpm for one hour at 16°C. After centrifugation, supernatant was removed until about 500μl above the Percoll-pellet still remained, which were collected.

### Transduction with papilloma PsVs

24h prior to transduction, 50,000 HEK293TT cells per well were seeded in a 24-well plate, or 8,000 HEK293TT cells per well were seeded in a 96-well plate.

PsVs were added directly to cell culture media. When gaussia luciferase was used as reporter, 20μl of cell culture supernatant was collected and stored at -20°C until the luciferase-assay was performed to check for free luciferase present in the PsV suspension.

In experiments with ι-carrageenan (Sigma-Aldrich), ι-carrageenan dissolved in PBS was added to cell culture media at the indicated concentrations immediately prior to addition of PsVs. All transduction experiments were performed in triplicates.

### Quantification of transduction

The *Gaussia* luciferase assay was performed 72h after transduction by transferring 20μl of cell culture supernatant into a black luminometer plate (NUNC) [19]. The substrate was prepared by 1:1,000 dilution of 2mM coelenterazine (P.J.K.) in assay buffer (1.1M NaCl, 220mM K_2_HPO_4_/KH_2_PO_4_, 0.44mg/ml BSA, 1.3mM NaN_3_, pH 5). Centro XS³ LB 960 Microplate Luminometer (Berthold, Bad Wildbad, Germany) was used to inject 100μl of the substrate, and measurement of relative light units was performed for 1sec after a delay of 1sec after injection for each individual well. Background measurements of cell culture media removed directly after transduction were subtracted from measurements 72h after transduction.

Firefly luciferase assay was performed 72h after transduction by lysing the cells for 2mins by adding Bright-Glo reagent (Promega). The measurement was carried out using a Centro XS³ LB 960 Microplate Luminometer within 5min after addition of the reagent.

When GFP was used as reporter, GFP-positive cells were counted 72h after transduction and transducing units per ml were calculated.

### Western blot analysis

20μl of each fraction was mixed with β-mercaptoethanol-containing loading buffer, and proteins were separated by SDS-PAGE. After blotting onto a nitrocellulose membrane, membranes were blocked with 5% non-fat dry milk in PBS-T (PBS containing 0.1% Tween-20) and incubated over night with MD2H11 antibody. MD2H11 is directed against a conserved sequence in human papillomavirus capsid protein L1. After incubation with the secondary antibody (polyclonal sheep anti-mouse IgG (H+L), peroxidase-conjugated, Jackson Immuno), the signal was detected by chemiluminescence with ECL substrate (Pierce) using the Intas Advanced Fluorescence Imager.

### Extraction of DNA from PsVs and quantitative PCR

10μl of pseudovirus samples were subjected to DNA-digest following the manufacturer’s instructions. The samples were incubated with 4 units DNaseI (NEB) for 60min at 37°C to remove any residual DNA that may still be present. DNaseI was heat inactivated for 30min at 75°C before DNA was extracted using the Qiamp MinElute Virus Spin Kit (Qiagen). DNA was eluted in 100μl, of which 5μl were used per reaction in qPCR. QuantiNova SYBR Green PCR Kit (Qiagen) was used for PCR reaction with the following primers for GFP: 5' ATC CTG GTC GAG CTG GAC GG 3' (forward) and 5' GAC GTA GCC TTC GGG CAT GG 3' (reverse).

In order to quantify the extracted DNA, a standard curve was created by diluting pEGFP plasmid to contain 3x10^5^ to 30 copies per reaction.

### Particle-Associated-Nucleic Acid PCR

Particle-Associated-Nucleic Acid (PAN)-PCR was performed according to the published protocol [20] after extraction of the DNA from PsV particles, as described above. Briefly, degenerated primers were used to amplify random DNA sequences present in the nucleic acid sample in a PCR. The resulting amplicons were cloned in to a pCR4-TOPO vector by TOPO-TA cloning (Invitrogen) according to the manufacturer’s instructions. After transformation of these plasmids into DH5α E.Coli, propagation and plasmid extraction, plasmids were sent for sequencing (Eurofins genomics, Ebersberg, Germany).

### Transmission electron microscopy

Density gradient ultracentrifugation purified VLPs were fixed for 24h with 2% formaldehyde in the presence of 0.05M HEPES. Thereafter, 3μl of the suspension were transferred to formvar/carbon-coated copper grids, dried for 20sec, washed with H_2_O and stained with 1% phosphotungstic acid (pH 7.0). The grids were observed with an EM 900 transmission electron microscope (Carl Zeiss Microscopy, Oberkochen, Germany) operating at 80kV. Micrographs were taken with a Variospeed SSCCD camera SM-1k-120 (Tröndle, Moorenweis, Germany).

### Mice

9–12 weeks old female BALB/c mice were obtained from in-house breeding. Mice were kept in isolated ventilated cages with unrestricted access to water and rodent chow.

All animal experiments were carried out in accordance with the EU Directive 2010/63/EU for animal experiments and were approved by local authorities (No.: TVV 49/15; DD24-51-31/331/52; Regional Council of Leipzig, Landesdirektion Sachsen, Referat 24, Germany).

For *in vivo* transduction experiments, 50μl of PsV suspension were injected into the left thigh muscle under inhalative isoflurane anesthesia. Mice never showed any signs of adverse reaction or inflammation at the site of PsV injection.

### Bioluminescence imaging

200μl of d-luciferin (15mg/ml in PBS) were injected intraperitoneally after inhalative isoflurane aesthesia of the mice. 20min after injection, luminescent images were acquired (1min exposure, medium binning and f/1) using an IVIS SPECTRUM (Xenogen, Perkin Elmer).

### Statistical analysis

Statistical analyses were performed using GraphPad Prism6. Differences were regarded as significant for p<0.05. Statistically significant differences are indicated as follows: * = p<0.05, ** = p<0.01, *** = p<0.001, ns = not significant.

## Results

### Analysis of ten different non-human papilloma PsVs

**Fig 1:**
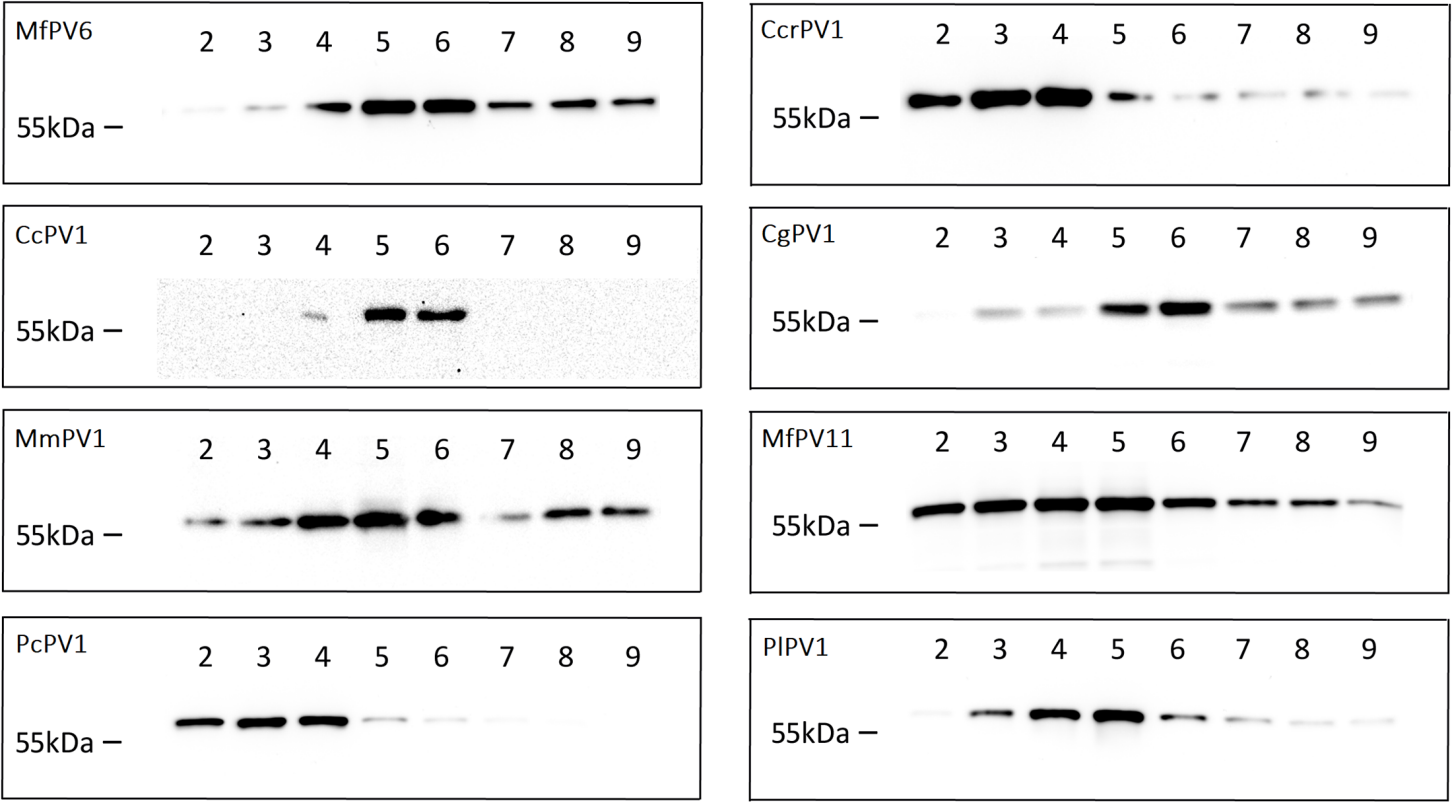
Western Blots of fractions after purification by ultracentrifugation. All produced non-human papilloma PsVs were purified by density gradient ultracentrifugation. Subsequently, the collected fractions were separated by SDS-PAGE, blotted onto a nitrocellulose membrane and probed for L1 using the MD2H11 antibody.

**Fig 2:**
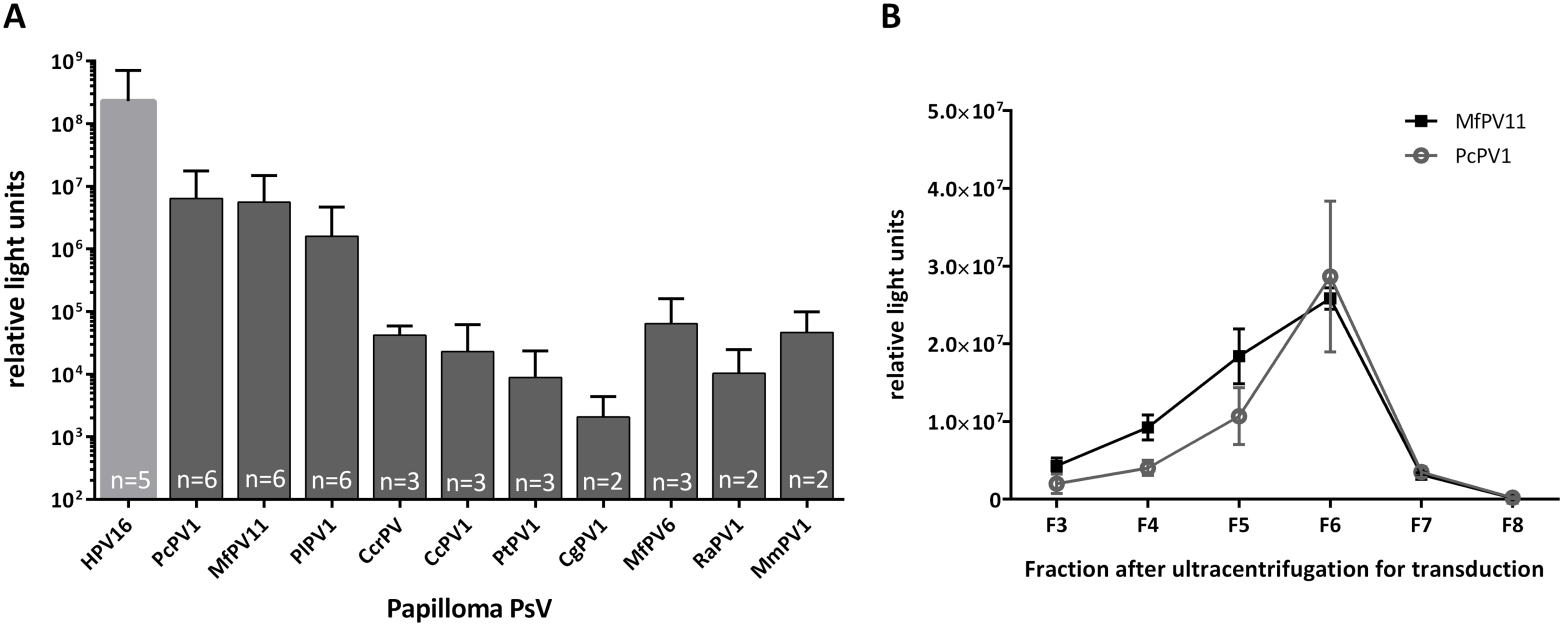
Luciferase assay 72h after transduction of HEK293TT cells with different non-human papilloma pseudovirions (PsVs). 50.000 HEK293TT cells were transduced by adding 10μl of fractions 3 to 8 of ultracentrifugation-purified papilloma PsVs. Cell culture supernatant was used for luciferase assay 72h after transduction. (A) Relative light units (RLU) measured after transduction with at least two independent PsV preparations as indicated. Shown are mean RLU values and standard deviations of the fractions yielding the highest transduction of one preparation. The background level measured directly after transfer of supernatant (0 hours) was subtracted from the 72 hours measurements for each preparation. (B) RLU for fractions 3–8 after transduction with PcPV1 and MfPV11 PsVs, presenting the typical peak of transducing PsVs around OptiPrep fraction 6. Shown are mean values and standard deviations of triplicates of one representative experiment.

**Fig 3:**
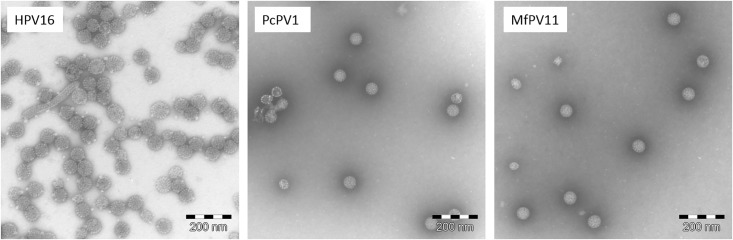
Transmission electron microscopy of HPV16, PcPV1 and MfPV11 VLPs. Purified VLPs were fixed for 24h at room temperature with formaldehyde, contrasted with phosphotungstic acid and analyzed by transmission electron microscopy.

The aim of this study was to explore a range of non-human papillomaviruses (NHPVs) for their suitability as gene carriers. Sequences for capsid proteins L1 and L2 of ten different papilloma viruses were identified in NCBI’s GenBank database (Table 1, abbreviations as published [17]). Based on the successful application of genus α-HPV PsVs as gene delivery vectors [12,15], we selected five of the ten analyzed NHPVs from the group of α-papillomaviruses, which naturally infect non-human primates (Table 1). After synthesis of the codon-optimized DNA-sequences and cloning into pcDNA3.1+ expression vector, VLPs were produced in HEK293TT cells and purified by OptiPrep density gradient ultracentrifugation. First analyses were performed by western blotting of the resulting fractions to check for the expected pattern, in which the majority of the purified VLPs would be found in fractions 4–6 due to their size and molecular weight. Eight of the ten NHPV VLPs were detectable by western blot (Fig 1 and Table 1) in the expected fractions. PtPV and RaPV VLPs were neither detectable by western blot nor in silver stained protein SDS-PAGE (not shown). In order to test the functionality of the NHPV PsVs as gene carriers, PsVs carrying pCMV-G.Luc as reporter plasmid were produced and used to transduce HEK293TT cells. 10μl of each fraction after ultracentrifugation were used to transduce 50,000 HEK293TT cells in a 24-well plate. Transduction efficiency assays revealed at least some low level of G.Luc expression after transduction for all tested PsVs, with the exception of PtPV. For the analysis of the transduction efficiency, RLU-values measured in the cell culture supernatant immediately after addition of the PsV suspensions were taken into account as background caused by free G.Luc. These values were subtracted from RLU values measured 72h after transduction. A transduction was only considered successful if the measured RLU-values after 72h were at least 100 times higher than the G.Luc background. G.Luc expression—as indirect measure for transduction efficiency—differed substantially between the individual papillomavirus types (Fig 2A). Remarkably, all tested NHPV PsV preparations showed transduction rates that were at least 10-fold lower than HPV16 PsVs. This and the higher amount of VLPs observed by TEM imaging (Fig 3) suggest that HPV16 generally yields higher amounts of PsV particles. Although TEM can strictly not be considered a quantitative method, all three preparations shown in Fig 3 were produced in parallel under the same conditions and therefore allow some quantitative insight. Of all NHPV PsV, PcPV1 and MfPV11 proved to be the types that not only led to the highest transduction rates, but can also be produced very reliably. Thus, in the following experiments PcPV1 and MfPV11 were analyzed further. Transduction experiments with PcPV1 and MfPV11 PsVs using HeLa-T, HEp2 and Vero cells showed expression of the reporter protein as well, but to a much lower extent than HEK293TT cells. NIH-3T3 and A549 cells did not show any transduction (data not shown).

**Table 1:** Non-human papilloma viruses selected for this study. Animal papilloma viruses that were produced as VLPs and PsVs.

Papilloma virus	Classification	Abbreviation	GenBank-Nr.
**Caretta caretta papillomavirus type 1**	Dyozeta 1	CcPV1	NC_011530
**Colobus guereza papillomavirus type 1**	Alpha 14	CgPV1	GU014532.1
**Common chimpanzee papillomavirus type 1**	Alpha 10	PtPV1	AF020905.1
**Crocuta crocuta papillomavirus type 1**	Lambda	CcrPV1	NC_018575
**Macaca fascicularis papillomavirus type 6, isolate Mac39**	Alpha 12	MfPV6	EF558840.1
**Macaca fascicularis papillomavirus type 11, isolate Mac1637**	Alpha 12	MfPV11	GQ227670.1
**Procyon lotor papillomavirus type 1**	Lambda 4	PlPV1	NC_007150
**Puma concolor papillomavirus type 1**	Lambda 1	PcPV1	AY904723
**Rhesus papillomavirus type 1b, isolate Mac170**	Alpha 12	MmPV1	EF591300.1
**Rousettus aegyptiacus papillomavirus type 1**	Psi 1	RaPV1	NC_008298.1

### PcPV1 and MfPV11 as gene vectors

In order to confirm the formation of papillomavirus capsid structures, PcPV1 and MfPV11 VLPs were produced and purified by OptiPrep ultracentrifugation. Electron microscopic analysis revealed that PcPV1 and MfPV11 indeed form spherical capsids with a diameter of approx. 60nm resembling human papilloma virions (Fig 3) as published previously [21,22].

**Fig 4:**
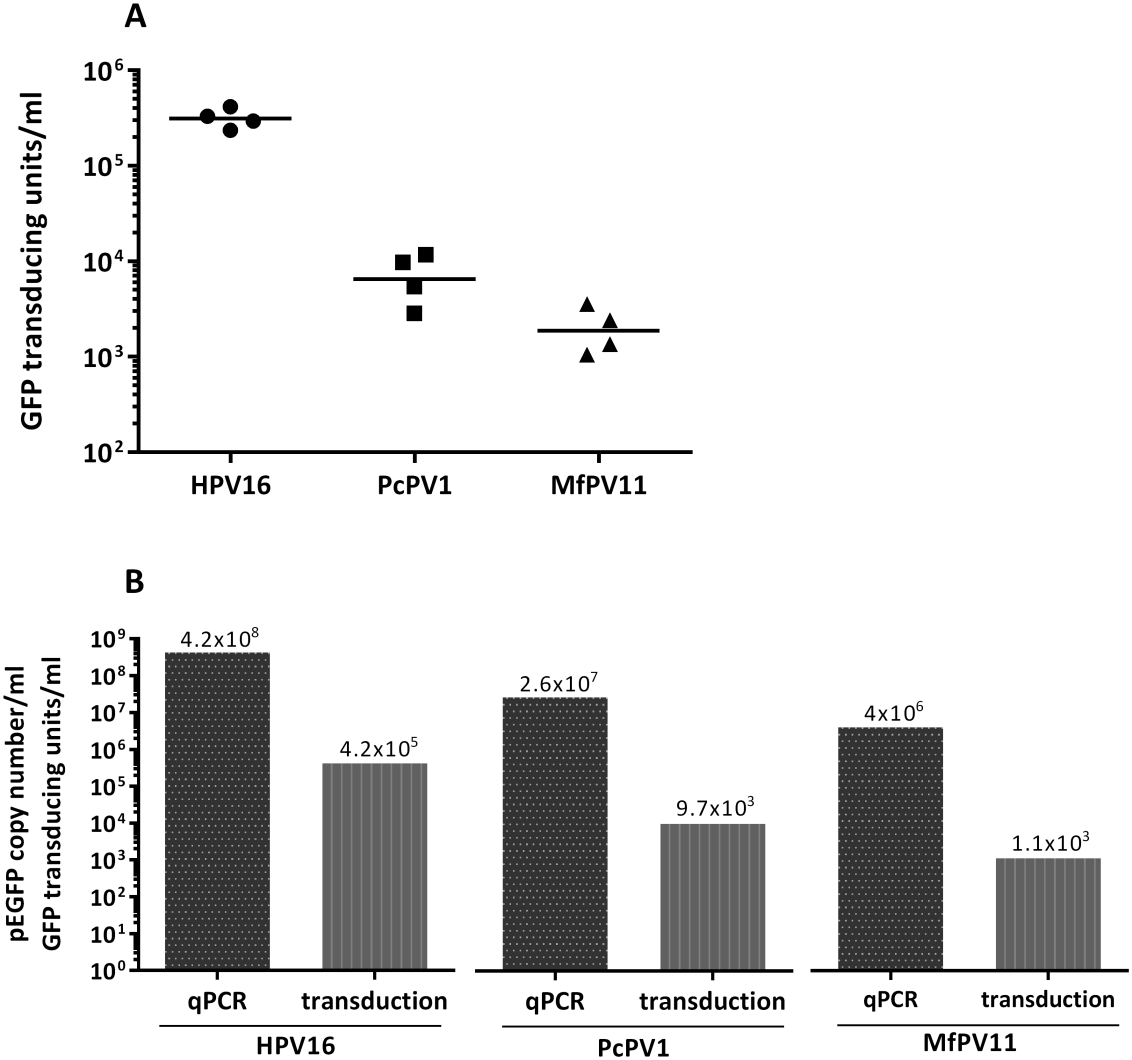
Titration of PsVs by transduction of HEK293TT cells. (A) HEK293TT cells were transduced with the indicated PsVs carrying pEGFP as reporter plasmid. 72h after transduction, GFP-positive cells were counted and titer was calculated as transducing units per ml PsVs. Each data point represents one PsV preparation. (B) To compare the amount of transducing units with the amount of particles carrying the pEGFP reporter plasmid, plasmid DNA was isolated from PsVs and quantified by qPCR to obtain the pEGFP copy number in the sample.

As transduction with the pCMV-G.Luc reporter plasmid provides an indirect measure of transduction efficiency, we repeated transduction experiments with PcPV11 and MfPV11 PsVs carrying pEGFP as reporter plasmid to determine transducing units by quantifying GFP-positive cells (Fig 4A). 72h after transduction, MfPV11 PsVs yielded titers of approx. 1.9x10^3^ transducing units/ml and PcPV1 PsVs of approx. 6.5x10^3^ transducing units/ml. Additionally, the packaged DNA was extracted from PsVs and the pEGFP plasmid was quantified by qPCR using EGFP-specific primers. The titer expression in pEGFP-plasmids per ml is based on the assumption that one plasmid is packaged per PsV particle (Fig 4B). Analysis by qPCR revealed a drastic difference between the amount of PsVs with a packaged reporter plasmid and the amount of transducing units, leading to the conclusion that not the number of produced particles but rather the efficiency of transduction, including the attachment or entry step, is the limiting factor when using papilloma PsVs for gene transfer.

**Fig 5:**
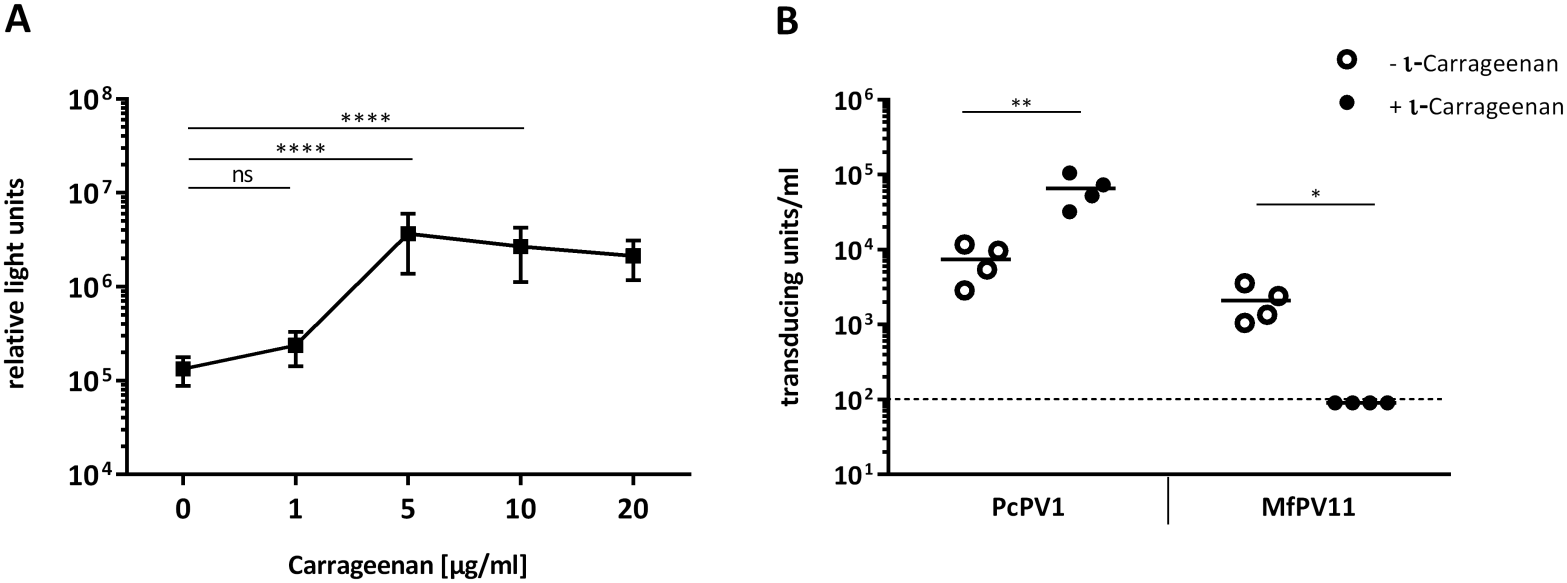
Effect of ι-carrageenan on transduction with PcPV1 and MfPV11. (A) Different doses of ι-carrageenan were added to the cell culture medium immediately before transduction of HEK293TT cells with PcPV1 PsVs carrying a G.Luc reporter plasmid. Luciferase assay was performed 72h after transduction. Shown are mean values and standard deviation of three independent PcPV1 PsV preparations. Statistical analysis was performed by 2way ANOVA and Tukey’s multiple comparison test. (B) Additionally, HEK293TT cells were transduced with PcPV1 and MfPV11 PsVs carrying a pEGFP reporter plasmid with (+) and without (-) addition of 10μg/ml ι-carrageenan. GFP-positive cells were counted 72h after transduction and transducing units were calculated. Each data point represents one experiment with one PsV preparation. Dashed line indicates limit of detection. Statistical analysis was performed using t-test.

In order to further characterize PcPV1 and MfPV11 PsVs, we tested the ability of ι-carrageenan, a sulfated polysaccharide, to influence the transduction *in vitro*. Ι-carrageenan has been described previously as a powerful inhibitor of genus α-HPV infection by preventing the virion from binding to the cell surface [23]. While ι-carrageenan did indeed prevent transduction with MfPV11 PsVs when it was added to cell culture medium together with the PsVs, the observed effect for PcPV1 PsVs was the exact opposite (Fig 5B). When ι-carrageenan was added to PcPV1 PsVs upon transduction, the expression of the G.Luc reporter protein significantly increased (Fig 5A). This experiment was repeated with pEGFP as reporter plasmid, which confirmed that the observed effect is due to a larger number of transduced cells and not to an increased amount of expressed reporter plasmid (Fig 5B). A similar ι-carrageenan induced increase in transduction efficiency was also observed for PlPV1, CcrPV1 and MmPV1 PsVs, while transduction with MfPV6 PsVs was inhibited (data not shown).

### MfPV11 and PcPV1 as gene carriers in vivo

**Fig 6:**
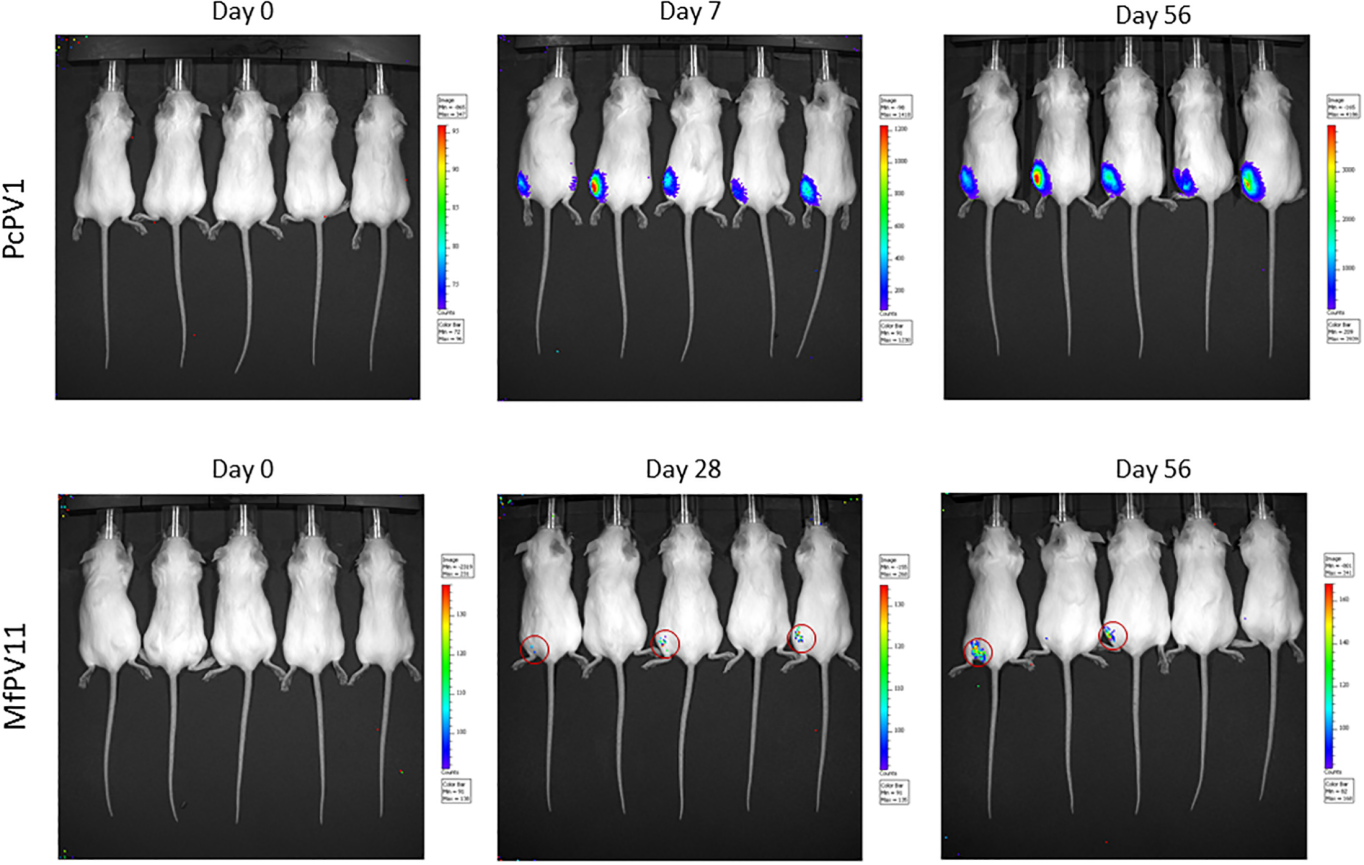
Bioluminescence imaging after intramuscular application. 50μl of the indicated PsV preparation were injected into the left thigh muscle. Bioluminescent imaging was performed approx. 3h after injection (day 0) to test for any free F.Luc and subsequently in a weekly manner.

It is worth noting that *in vitro* transduction with PcPV1 and MfPV11 PsVs has only been moderately effective on any tested cell lines other than HEK293TT in our hands. Therefore, the most pressing question was whether transduction *in vivo* would be observable. As a simple way to assess gene transfer by PsVs and subsequent protein expression *in vivo*, we chose firefly luciferase (F.Luc) as a reporter. MfPV11 and PcPV1 PsVs carrying an F.Luc reporter plasmid were produced without purification by ultracentrifugation, 2μl were used to transduce HEK293TT cells and F.Luc assay was performed 72h after transduction. Both PsV preparations showed transduction at similar levels with 3.09x10^6^ RLU/ml for PcPV1 and 4.71x10^6^ RLU/ml for MfPV11. 50μl of each PsV suspension were injected intramuscularly into the left hind leg of female BALB/c mice. Approx. 3h after application, the mice were subjected to bioluminescent imaging in order to check for any free F.Luc that may have been present in the PsV preparation (Fig 6 “Day 0”). Mice were then monitored on a weekly basis by bioluminescent imaging (Fig 6). Mice that received PcPV1 PsVs showed a pronounced expression of F.Luc 7 days after application, while it took 28 days until a weak F.Luc signal was detectable in mice who had received MfPV11 PsVs. The F.Luc signal in the PcPV1 group remained detectable until at least 10 weeks after application, which is when the last bioluminescent imaging was performed.

### Analysis of DNA found inside the PsV particles

One major concern with the use of papillomavirus based vectors for gene transfer is the possibility of unspecifically packaged chromosomal DNA from the produced cells, which may be found inside the particles. To address the question of what type of DNA is present in PcPV1 and MfPV11 PsVs, DNA was extracted from PsV preparations and amplified by PAN-PCR [20]. Sequencing of the resulting amplicons revealed the presence of both plasmid and human chromosomal DNA. Although quantitative conclusions are not possible using this method, we have to assume that the production of PcPV1 and MfPV11 PsVs in cell culture will yield a heterologous batch of PsV particles. While most particles will likely contain plasmid, some will have encapsidated cellular DNA. This is in line with previous reports stating that a fraction of papilloma PsVs produced in cell culture will indeed encapsidate cellular DNA [21].

## Discussion

Though not yet a competitive alternative to currently approved protein-based vaccines, gene-delivery through viral vectors holds great potential to increase the applicability of genetic vaccines. Due to the large amount of currently known NHPV types, these viruses provide the possibility to develop a large viral vector platform. Although cross-neutralization between different PV types is theoretically not expected, it remains to be elucidated in future studies to which degree cross-neutralization between antibodies developed by HPV- or NHPV-vaccinated individuals may restrict the gene transfer by NHPV PsVs. In this study we examined a range of NHPVs for gene delivery in cell culture and identified PcPV1 PsVs as effective for intramuscular delivery of a reporter gene. Gene sequences for the capsid proteins L1 and L2 of more than one hundred animal papillomaviruses are available in GenBank and can be used quite easily to produce pseudovirions. A main difficulty lies in the limited predictability of the suitability of the various types of PsVs to transduce a plasmid of interest: not only did we observe substantial differences in the transduction efficiency *in vitro*, but transduction *in vivo* must be tested for every individual papillomavirus type. Since transduction *in vitro* and *in vivo* has been shown for various PsVs based on genus α-HPVs [21], we speculated that this genus might provide especially suitable candidates for gene delivery. Interestingly, this did not prove to be the case. Of all tested PsVs of non-human α-papillomaviruses (CgPV1, PtPV1, MfPV6, MfPV11, MmPV1) only MfPV11 yielded good *in vitro* transduction rates. Upon intramuscular injection, however, MfPV11 PsVs showed only very week or no detectable transduction efficacy of the reporter plasmid and subsequent F.Luc-expression *in vivo*. A large number of HPV types belong to the genus of mucosa-infecting α-papillomaviruses, and α-HPV PsVs have indeed been successfully used for genital transmission in mice [21]. Although this approach shows promise for mucosal application of papilloma PsVs as gene vectors, intense physical pretreatment of the mucosa was necessary, as the intact mouse genital epithelium was found to be quite resistant to infection with HPV16 PsVs [12,24]. The tropism of native papillomaviruses is thought to be primarily dictated by tissue specific enhancers, rather than by specific cellular entry receptors [25,26]. As many cell types have been shown to be susceptible to PV infection [27], it is therefore possible that the natural PV tropism has no bearing on *in vivo* transduction with PsVs. Intramuscular injection is a well-established and commonly accepted route for vaccine administration. We show here that this form of application could be a simple alternative for the administration of papilloma PsVs for gene transfer. As the PsVs were injected into the muscle, we assume that mainly muscle cells were transduced and expressed the reporter gene, although we did not check in detail, which cell type was transduced. Luciferase signals were observed only at the site of injection, but it would be of great interest to analyze whether an application to other organs would also be feasible.

Furthermore, it would be interesting to analyze the use of ι-carrageenan as additional transduction enhancer *in vivo*. ι-Carrageenan has previously been shown to be a potent inhibitor of genus α-HPV infection by preventing the virions from binding to cells. To our knowledge, it has not been previously published that for certain papilloma virus types the effect of ι-carrageenan can be just the opposite, leading to a significantly increased transduction *in vitro*. Whether this is also the case *in vivo* remains to be elucidated. The observation that injected carrageenan causes inflammation [28,29] could even be explored as a potential adjuvant. Studies analyzing the attachment mechanisms of HPVs have shown that on epithelial cells, HPVs use heparan sulfate proteoglycans (HSPGs) as primary attachment factors [30–32]. This finding is supported by the fact that infection with α-HPVs can be blocked by heparin or other sulfated polymers like carrageenan [23,31]. Interestingly, the sensitivity to ι-carrageenan differs substantially from papillomavirus type to type. Bovine papillomavirus type 1 and cottontail rabbit papillomavirus have been shown to be 100 times less sensitive than α-HPVs, while HPV5—a β-papillomavirus—did not show any inhibition by ι-carrageenan [23]. In case of the keratinocyte cells HaCaT, the infection with HPV-16 was shown to increase at higher heparin concentrations. This effect was especially pronounced in HSPG-deficient cells [33], showing the ambiguous effect that sulfated polysaccharides can have depending on the cell type or papillomavirus type. Little is known about a potentially different entry pathway employed by the non-human papillomaviruses used in this study. The mechanism behind ι-carrageenan mediated enhancement of transduction that we observe for certain papillomavirus types is therefore unknown at this point.

Apart from a mucosal application, it might be worth exploring the (sub- or trans-) cutaneous route for PsV administration. In the context of genetic vaccine applications, the skin is an attractive target due to the presence of Langerhans and dendritic cells as antigen presenting cells [34,35], which HPVs have been shown to be able to enter [36].

In conclusion, we show that PcPV1 PsVs effectively transduce *in vitro* and—more interestingly–*in vivo* after application into the muscle, leading to a several week-long expression of a reporter plasmid. The vast amount of known and sequenced non-human papillomaviruses bodes well for their possible application as gene vectors. Circumventing the safety issue of the PsVs encapsidating chromosomal DNA is a crucial step in order to move forward towards preclinical and clinical applications. Alternative protocols have been suggested for PsV production to eliminate the packaging of any DNA other than the desired plasmids [37]. Even more promising is the production using cell-free production systems in the absence of chromosomal DNA, which might fulfill the requirements for good manufacturing practice (GMP) [21]. We therefore assume that NHPVs-based PsVs are potentially suitable as a gene transfer platform for further *in vivo* studies.

## Supporting information

S1 File Codon-optimized sequences for non-human papillomavirus capsid proteins L1 and L2. (PDF)
