# Supplementary material for: Non-human papillomaviruses for gene delivery in vitro and in vivo
Source: PLoS One. 2018 Jun 18;13(6):e0198996. doi: 10.1371/journal.pone.0198996 (PMC6005490; doi:10.1371/journal.pone.0198996)
Supplement: S1 File — (PDF) [file pone.0198996.s001.pdf]

S1 supporting information

Codon-optimized sequences for non-human papillomavirus capsid proteins L1 and L2

CcPV1

L1

ATGGCCGTGTGGACCCCTAGCACCAAGGCCCTGTTTGTGCCCCCCGTGAATGTGCCCACCCTGTACAGCACCCG
GGAATACGTGCGGCGGACCAGCTATGTGTTCCACGGCACCACCGAGCGGCTGATCACCATCGGCAACCCCTAC
TTCGCCCTGACCGACAACGCCACCGTGACCGTGCCTAAGGTGTCCGCCTACCAGCACCGGGTGTTCCGGATCA
AGCTGCCCGACCCCAACAAGTTCCCCATCCCTGAAAGCGCCGTGGGCGACAGAGACACCACCAGACTTGTGTG
GGCCGTGCGGGGCATCCAAGTGAACAAGTCTCAGCCTCTGGGCGTGGGCGCCAGCGGCAACACCATGTTTAA
CGGCCTGCAGGACTTCGCCGAGACACACCACCCCAGCATGGAAAAGCCCGACCCTCCCGAGGACAGAAGAGT
GAACGCCGCCTTCGACGCCAAGCAGAGCCAGGCACTGATCGTGGGCTGCATCCCTCCTGTGGGCCAGCATTGG
GATGCCGCCAAGAGATGCGTGGAAGATAACAACAAGGACATGTGCCCTCCACTGGAACTGCAGCACACCGTG
ATCGAGGACGGCGACATGATCGACATGGGCATGGGCACCCTGAACTTCAAGAGCCTGAGCCTGAACTGGTCC
ACCCTGCCCCTGGAACTGATCAACAGCGTGTCCAAGTACCCCGACTGGCTGACCATGAACGCCGACCCCTACG
GCAACCACTGCTTCTTCATGCTGAAGCGGGAACAGGTGTACATGAAGGGCGTGGGACTGCACCTGGGCAACA
TCGGCGAGGATGAGCCCACCACCATGTTCCGGAAGGGCACCACAGGCCAGAAGTACCAGACCCCCGGCAGAC
ACAGCTGGTTCCCTCTGCTGAGCGGCAGCCTGAGCACCAGCGACAATCAGCTGTTCAACCGGCCCTACTGGCT
GGAAAACAGCACCGCCCCCAACGACGGCATCTGCTGGCACAACCAGATGTTCGTGACCTGCGTGGACACCACC
CGGAATACCATCTTCCAGATCAGCCAGTTCAAGAAAGGCGTGACCGCCACCGCCGACTACAAAGAGGCCAACT
ACGATATGTACGCCCGGCACGTGGAAGAGTACGAGATCAGCTTCATCCTGCAGCTGTGCAGCATCAAGATGG
ACCTGCCCGTGCTGAACCATCTGCACAACATGGACGCCAGCCTGCTGGACGATTGGGGCTTTGGAGCCACCCC
CCCTCAGAACCTGACTGTGGAAGATCAGTACCGGTTCCTGAACAGCAAGGCCACCAAGTGCCCACCTCCACCA
GCCACACCAGCCGATGCTGATCCCTGGGGCAAGTACAAGTTCTGGGACGTGGACTGTACCGCCCAGATCTCCA
GCGACCTGACCCCCTTTCCTCTGGGACGGCGGTTCCAGCAGCTGTATCCTCAGGCCGGAAAGCCTGCCCCCAG
CAACCCTAGAAAGCGGCGGAGAGGCAGATGA

L2

ATGATGAGCAAGCGGCGGAGAGTGACCAGAGCCAGCCCCGATGACATTTGGCGGCACTGCAAGCAGTTCGGC
GACTGCCCCGACGACATCCAGAAGGTGTACACAGGCAACACAATCGCCGACAACATCCTGAAGTGGGCCAGC
AGCTTCCTGTTCTTCGGCGGCCTGGGAATCGGATCTGCCGAAGGCGCTGTGGCCGCTGCCGCCTCTGAACACA
TCCTGCCTATCGGCGGAGGCAGCCTGCCCAAGCAGCCTATCGATGTGCCCATCACCAGAGTGCCCGCCAGCAA
TGTGACCCCCGGCTTCAGCGACATCACCGTGAACCCTGACGTGGCCCTGGATGCCGGAACAGTGGTGCATGCC
GCCGAACCTGTGGATCCTGTGTCTGGCACCCCCCCCATCATTCACGCCAGCCCCAATAGCACCGAAGTGATCCC
CCCTATCCGGCCCGTGGAAAACCCCCCCTGGCAGAACCCTTTCGACAGCGGCCTGGAAACCCCAGGCGTGAAC
GTGGGCGTGGTGGATTACAGCGCCGGCAACGAGATCGAGCTGAGCGTGCTGTCTAGCACCGCCCCTACCCTG
ACCAACGCCGTGGAAGAGACAGAGCTGTTCAGCAGATTCGAGCTGGACCCCAGAACCAGCACCCCCAACACC
ACAACAAGAGGCGGCTGGATGTCCCACGTGGCCGTGGGCAGATTTGCCAAGACAGCCGCCAGAGAAGTGCCC
CTGCCTGTGCTGACATCTACCGGCGGAGTGATGCAGTTCGAGAACCCCGCCTTCGAGTTCAGCGAGGCCGTGT
CCGAGGTGTCCCGGTCCATCAGCTTCAACGACCCCGACAGCGCCCCCTTCGCCAGACTGTCTAGACCCAGCCTG
TTCCAGAGAGCCGGCAGACTGGGAGTGCAGAGAGTGGGCAATCTGCTGGGCATGGTCACCAGGGCCGGCAA
GCAGCTGTTCGTGCCCCGGGTGTACTACAACGAGCTGTCCAGCATCTTCGAGAGCCCCGACGTGCTGGAAATG
GAACCCATCATCATCGAGGACAGCGGACCCCCCATCGAGGATGAGGCTATTCCTGGCGCTCCTGCCGGCGTGT
TCCCACAGGGCAATAGACCCTACGCCTACAACGGCTACCTGTTCGGCCCCATCCCCGTGGACGTGTCCATCAAG
GTGTCCGGCACCGGCTTCATCCCCATGCCTGTGACCGTGTCCGGAAACACCATCTTCCCACTGTACCCCAGCTTC
GACAAGAGCACCCCCCTGTACCCCCCCAGACACGTGTTCTTCTCCGACCTGGACGACCCCATCATGTTCAAGCG
GAGAAAGAAGTGCTTCGCCGACGGCTGCGTGGACGCCTTCTACTGA

<u>CcrPV1</u>

L1

ATGGCTGTGTGGCTGCCCGCCCAGAACAAGTTCTACCTGCCCCCTCAGCCCAGCACCAGAGTGCTGAGCACCG
ACGAGTACGTGACCCGGACCAGCATCTTCTACCACGCCAGCACCGACCGGCTGCTGACAGTGGGCCACCCCTA
CTTCGACATCTACCACGAGAACAAGAAAGACATCATCGTGCCCAAGGTGTCCCCCAACGCCTACCGGGTGTTC
AGACTGAAGCTGCCCGACCCCAACAACTTCGCCTTCGGCGACAAGAGCATCTTCGACCCCGAGAAAGAACGGC
TCGTGTGGGCCCTGAGAGGCGTGGAAATCGACAGAGGCCAGCCTCTGGGCTGTGGCATCACAGGCCACCCCA
TCTTCAACAAGTTCGCCGACGTGGAAAACGCCAAGAACGTGGGCACCGGCCACGACGCCATGAATGCCATCG
GCAGCAACACCGCCTTCGACCCTAAGCAGACCCAGATGTTCCTGATCGGCTGCAAGCCTGCCCTGGGCGAGCA
TTGGTCTAGAGCCGCCTGGTGCAAGAACAACGAGGGCGGAGGACTGGGCCACAAGGACACCGATTGCCCCCC
CATCGAGCTGAAAACCACCAGCATCGAGGACGGCGACATGGTGGACATCGGCTTCGGCGCCATGGACTTCAA
CGACCTGCAGCAGGACAAGACCAGCGTGCCCCTGGACATCTACAAGAGCAAGTGCAAGTACCCCGACTACATC
AAGATGGCCAACGACCCCTACGGCGACTTCTGCTTCTTCTACGTGCGGCGCGAGCAGATGTACGCCCGGCACT
ACTTCACCAGATACGGCAAGATCAGCGAGAAAGAGCAGGGCGACACCCTGGAAGATGACAACCCCCCCCTGT
CCACCAACAACTACTTTACCAGCCCCAGCGGCAGCCTGGTGTCTAGCGAGGGCCAGCTGTTCAACCGGCCCTA
TTGGATCCAGCGGAGCCAGGGCCAGAACAACGGAATCGCCTGGAACAATCAGCTGTTCCTGACCGTGGTGGA
CAACACCAGAGGCACCGCCCTGAATATCATCGTGGGCCAGAATGGCACCCCCAACCAGGGCGCCTTCAAGGCC
AACGAGTACTACACCTACCTGCGGCACGTGGAAGAGTTCGACATCAGCGTGATCCTGCAGCTGTGCAAAGTGC
GGCTGACCCCCGAGAACCTGGCCATCATCCACACCATGGACCCTAACATCATCGAGGCCTGGCACCTGAACGT
GAACCCCCCTTCCGGCATCCTGGACGAGACATACCGGTACATCCACTCTATGGCCACCAAGTGCCCCAGCAACG
TGCCCCCCAGCGAAAAAGAGGACCCCTACAGCAAGCTGAAGTTCTGGGAGGTGGACCTGCGGGACAGACTGA
CCGAGCAGCTGGATCAGACCCCCCTGGGCCGGAAGTTCCTGTTCCAGACCAACGTGATCAGAGGCGGCGTGA
AGCGGCCCAGAGTTGTGACCACAAGCAGCAAGGCCAAGCCCGTGAAGAGAAGGCGGGGCAACAAATGA

L2

ATGCACGCCAGACGGAAAAGAGCCGCCCCTAAGGACATCTACCCCAGCTGCAAGATCAGCAACAACTGCCCCG
ACGACATCCGGAACAAGATCGAGCACAACACCCTGGCCGACAAGATCCTGAAGTGGGGCTCTGCCGGCGTGT
TCTTTGGCTCTCTGGGCATCGGCACCGGCAGAGGCACAGGCTCTTATGTGCCACTGGGCAGCGGCGTGAACGT
GGGCACCAGAGTGTCTACCGTGAAGCCCAGCATCCCCATCAGCAGCGTGGGCACAGCCGACGTGATCCCCGT
GGACGCCATGAATCCTCTGGGACCTGCTCTGGCCCCTCCCAAGTTTCCTACCGCCGTGGAAGATCCCGTGATCA
TCAGACCCCCCAAGTTCCCCAGCATTGTGGAAGATCCTGTGATTGTGCACAGCGCCGCTGAGCACCCTGCCGT
GCTGGACCCTGATCACATTGCCGTGGTGGACATCAGCGGCGAGACAGTGCAGGAAATCCCCTACACCACCAGC
AACGTCGTGACCGAGGAACAGCCCGCTGTGCTGGACGTGTCCACCGAGACAAGAGCCCCCAAGATCATCAGC
CGGACCCAGTACGAGAACCCCAGCTTCGAGGTGGCCATCACCTCCAATGCCACAGCCGGCGAAACCAGCGCCA
CCGACCACATCCTGGTGGACGGCTATTCTGGCGGCCAGCACATCGGCGAGCAGATCGAGCTGCAGGAACTGG
CCAGACGGTCCTTCAGCACCACCATCGAGGAAGAAACCAGCTTCCTGACCAGCACCCCCAACGAAGCTGTCGT
GCGGCCCAAGACCCGGAACCTGAACAGCAGAAGATACCTGCAGACCCAAGTGACCGACCCCGCCTTCGTGAC
CCAGCCTAGAAGCCTCGTGACCTTCCAGAACCCTGCCTTCGACGAGAGCGTGGACCTGATCTTCGAGAAGGAC
GTGGCCGACTTCCCCCTGGCCGCTCCTAACGAGGACTTCCGCGATCTGATCAGCCTGAGCAAGCCCATCTACCA
CCGGTCCAACGAGAACACCGTGCGGGTGTCCAGATTCGGCACCAAGGCCAGCGTGAAAACCAGATCCGGCGT
GATCGCCGGACCCCAGATCCACTACTTCTACGACCTGAGCGAGATCGCCCCTGCCGACAACATCGAGCTGGCC
ACACTGGGCTCTAGCCCTGTGGGAGAGCAGAGCGGAGAGAGCGTGATCTCTAGCGGCACCACCGACATGGAA
ATCATCTCCCTGACCGGCAGCACCCTGGAAAGCTACTCCGATGAGAGCCTGCTGGATATCTACGAGCCTATCGC
CAACGACCTGCAGCTCGTGATCGGCATCGGAAGAAGAGTGCGGCCTATCAGCGTGCCCGACCTGCTGACCACC
AAGTTCCAGATCTTCCCTGGCTTCGAGGGCGTGCACGTGCACACCAGCAGCAGCAACGAGACACCCAAGATCC
CTATCAACCCCCTGGAAACCCCAGCCGTCGTGATTGATCTGCTGGGCGGCACCGACTTCTACCTGCACCCCGCC
CTGTTCAAGAAGAAGAAAAAGCGGCTGTTCTGCGACTTCTTCGCCGACGGCGGAGTGGCCTCCTGCACCGAAT
GA

CgPV1

L1
ATGGCTATGTGGCGGCCCAGCGACAACAAGGTGTTCCTGCCTCCTGCCACCCCCGTGTCCAAGGTGCTGAGCA
CCGACGACTACGTGTCCCGGACCACCATCTACTACTACGCCGGCAGCAGCAGACTGCTGACCGTGGGCCACCC
CTACTTCCCCATCAAGAAGTCCGGCGGCAGAAACAGCCTGCTGGTGCCTAAGGTGTCCGGCTACCAGTACCGG
GTGTTCAGAGTGCGGCTGCCCGACCCCAACAAGTTCGGACTGCCTGAGGGCAGCCTGTACAACCCCGAGACAC
AGAGACTTGTGTGGGCCTGCAGAGGCGTGGAAGTGGGCAGAGGACAGCCTCTGGGCGTGGGCACATCTGGC
CACCCTCTGAGCATCGACCTGGAAGATACCAAGAACAGCACCCTGTTCGATGGCGCCCCTGGCAACGACAGCA
GAGACAACGTGTCCATGGATTACAAGCAGACCCAGCTGTTCATCATCGGCTGCAAGCCCCCACTGGGAGAGCA
CTGGGCTAAGGGCACCCCCTGCAATAGCAGCACCGTGAACGCCGGCGATTGCCCACCTCTGGAACTGGCCAGC
ACCACCATCCAGGACGGCGACATGGTGGATACCGGCTTCGGCGCCATGGATTTCGCCGCCCTGCAGAGCAACA
AGAGCGACGTGCCCCTGGACATCCTGAACGCCACATGCAAGTACCCCGACTACCTGCAGATGGCCGCCGAGCC
CTACGGCGACAAGATGTTCTTTAGCCTGCGGCGCGAGCAGATGTTCGTGCGGCACTTCTACAACAGAGCCGGC
ACCATGGGCGAGAGCGTGCCCGAGGAACTGATCCTGAAAGGCGCCCCTAGCAGCTCCAGAGCCACACCTGGC
AGCTCCATCTACGCCAGCACACCCAGCGGCAGCATGGTGTCTAGCGAGAGCCAGCTGTTTAACAAGCCCTACT
GGCTGCAGCGGGCCCAGGGAAGAAACAACGGCATCTGCTGGGGCAATCAGGTGTTCCTGACCGTGGTGGAC
ACCACCCGGTCCACCAATCTGACCGTGTGTGCCACAGCCACCAGCGAGACAACCTACAAGGCCAGCAACTTCA
AAGAGTACCTGCGGCACGGCGAGGAATTCGACCTGCAGTTCATCTTCCAGCTGTGCGTCGTGAACCTGACCGC
CGAAGTGATGACCTACATCCACGGCATGGACCCATCCCTGCTGGAAGATTGGAACTTCGGCACCCTGCCCCCTC
CAAGCGCCTCTCTGGGCGATACCTACCGGTTCCTGCAGTCCCAGGCCATCACCTGTCAGAGGCCTCCAGCCCCC
GAGAAGGACAAGCAGGATCCTTACGCCGGCCTGACCTTCTGGGAGGTGGACCTGACCGAGCGGTTCAGCGTG
GACCTGGACCAGTTCCCCCTGGGCAGAAAGTTCCTGCTGCAGACAGGCGGCAGACCTAGAGCCGCCGCTGCC
AGCAGAAAAAGAACAGCCGCTCCTGCCGCTGCCCCTGCCCACAAGAGAAAAAAGACCAAGAGATGA

L2
ATGGTGGCCAGAGCCAGAAGAAGAAAGCGGGCCAGCGCCACCCAGCTGTACCAGACCTGTAAAGCCGCCGG
AACCTGCCCCCCTGACATCATCCCTAAGATCGAGCACAGCACCGTGGCCGACAACATCCTGAAGTACGGCAGC
CTGGGCATCTTCCTGGGCGGCCTGGGAATCGGCACAGGCTCTGGAACAGGCGGCAGAACCGGCTACATCCCC
GTGGGCTCTAGACCCCCTACCGTGGTGGATGTGGGACCCGTGGCTAGACCTCCCGTCGTGATTGAACCTGTGG
GCGCCAGCGACCCCAGCATCGTGACACTGGTGGAAGATAGCAGCATCATCCAGGCTGGCGCCGCTCACCCCAA
TTTCACAGGCAGCGGCGGCTTCGAAGTGACCACCAGCGGCACAACAACCCCTGCCGTGCTGGATATCACACCA
GCTGGCGGCGGAGTGCAGATCAGCAGCAGCTCCTTCAGCAACCCCCTGTTCACCGAGCCCCAGTTCGTGGAAG
CTCCCCAGACCGGCGAAGTGTCCGGCCACATCCTGATCAGCACCCCTACAAGCGGCGCTCACGGCTACGAAGA
GATCCCCATGGTCACCTTCGCTCAGGAAGGCTCCGGCCTGGAACCCATCAGCTCTACACCTCTGCCTGGCGTGC
GGAGACTGGCCGGACCTAGACTGTACAGCAGAGCCTACCAGCAAGTGCGGGTGGACGACCCTCAGTTCGTGT
CTCAGCCTGCCACCTTCGTGACCTACGACAACCCCGTGTACGACCCCGAGGAAACCATCCTGTTCGACCGGACC
GGCCTGCACGATCCCCCCGATCCTGACTTCCTGGACATCGTGGCCCTGCACAGACCTGCCCTGAGAGCCACAA
GACAGGGCAGCGTGCGGTTCAGCAGACTGGGCAGAAGGGCCACCCTGAGAACCAGAAGCGGCAAGACCATC
GGCGCCAGAGTGCACTTCTACCACGACCTGAGCCCCATCTCTGCCGCCGACAATATCGAGCTGCAGCCCCTGCT
GCCCGTGGATCCTTCTGGCGTGACACCCGACGAGCCTGTGTACGACATCTTCGCCGACCCCGATGCCCTGCAG
CAGGCTGCTCCAAGCCAGAGAAGCAGCCTGAGCGTGTACAGACCCAGCGTGGTGGCCCTGTCCGCCACAAGC
TCTCACCCTAGCACCGTGCCTCTGTCTGCTGGCGTGGACGCCCCTGTGTTTAGCGGCCCTGATGTGGATATCCC
TGGCGCCTCTCCATGGCCTCAGCCAGTGCCTCCTCACACCACACCCCAGCACAGCATCTACGTGCACGGCACCG
ACTTCTACCTGCTGCCAGGCTACCTGTTCGTGCCCAAGCGGCGGAAGCGGTTCATCTACAGCTTCGCCGATGGC
TACGTGGCCGCCTGA

<u>MfPV6</u>

L1

ATGAGCCTGTGGCGGCCCAGCGACAGCAAGGTGTACCTGCCTCCTGCCCCCGTGTCCAAGGTGGTGTCCACCG
ATGAGTACGTGACCCGGACCAGCATCTACTACCACGCCGCCAGCTCTAGACTGCTGGCCGTGGGCCACCCTTA
CTACGCCGTGAAGAAAGGCAACAAAGTGACCGTGCCTAAGGTGTCCGGCCTGCAGTACCGGGTGTTCAGAGT
GCGGCTGCCCGACCCCAACAAGTTCGGACTGCCCGATGCCAACTTCTACGACCCCAATACCCAGCGGCTCGTG
TGGGCCTGTATCGGCCTGGAAGTGGGCAGAGGACAGCCTCTGGGCGTGGGCACATCTGGACACCCCCTGCTG
AACAAGCTGGACGACACCGAGAACGGCCCCAAGGTGGCAGCTGGACAGGGCGTGGACAACCGCGAGTGCGT
GTCCATGGATTACAAGCAGACCCAGCTGTGCCTGCTGGGCTGCAAGCCTCCTGTGGGAGAGCACTGGGGCAA
GGGCAATCCTTGCTCTGCCGGAAACGCCGGCGATTGCCCTCCACTGGAACTGCAGAACAGCGTGATCCAGGAC
GGCGACATGGTGGATACAGGCTACGGCGCCATGGACTTCAGCGTGCTGCAGGCCAACAAGTGCGACGTGCCC
CTGGACATCTGCAACAGCGTGTGCAAGTACCCCGACTACCTGAAGATGGCCGCCGAGCCCTACGGCGACAGCC
TGTTCTTTTACCTGCGGCGCGAGCAGATGTTCGCCCGGCACATGTTCAACAGAGCCGGCAACATTGGCGACGC
CGTGCCCGACGAGCTGTACATCAAGGGCTCTGGACAGAAGGCCGCCCTGCCCAGCCACATATTCTTCCCAACA
CCCTCCGGCAGCATGGTCACCTCTGAGGCCCAGCTGTTTAACAAGCCCTACTGGCTGCAGCGGGCCCAGGGCC
ACAACAATGGCATCTGCTGGGGCAACCAGGTGTTCCTGACCGTGGTGGACACAACCCGGTCCACCAACATGAC
CCTGTGTGCCGCCACCGGCACCGACAGCACCTACAAGAACGAGAACTTCAAAGAGTACATGCGGCACGTGGA
AGAGTACGACCTGCAGTTCATCTTTCAGCTGTGCAAGATCACCCTGACCACCGAAGTGATGGCCTACATCCACA
ACATGGACGCCAGCATCCTGGAAGATTGGAACTTCGGGCTGCAAGCTCCCCCCAGCGGCTCTCTGCAGGACAC
CTACAGATTCGTGACCAGCAGCGCCATCACCTGTCAGAAGCCTGCCCCCCCTAAAGAGAAAGAGGACCCCCTG
GCCAAATACGCCTTCTGGGACGTGAACCTGAAAGAGAAGTTCAGCGCCGACCTGGACCAGTTCCCCCTGGGCA
GAAAGTTCCTGCTGCAAGCAGGCATGCGGGCCAGACCCACCCTGAAGAAGAGAAGCGCCCCTAGCACCAGCA
GCAGCACCCCCGCCAAGCGGAAGCGCGTGAAGAGATGA

L2

ATGAAGCACGCCCGGCTGAGCAGAAGAAAGCGGGCCACACAGCAGAGCCCCCCCAGAGCCAGAAGAAAAAG
AGCCAGCGCCACCCAGCTGTACCAGACCTGTAAAGCCGCCGGAACCTGCCCCCCTGACGTGATCCCTAAGGTG
GAAGGCAGCACCGTGGCCGACCAGATCCTGAAGTACGGCAGCATGGGCGTGTTCTTCGGCGGCCTGGGAATC
GGCACAGGCTCTGGAACAGGCGGCAGAACCGGCTATGTGCCTCTGGGCGCTAGACCCTCTGTGGTGCCTGAG
GTGCTGCCCAGACCTCCCGTGACAGTGGAACCTGTGGCCCCCACCGATCCCAGCATCGTGTCTCTGCTGGAAG
AGAGCAGCCTGATCGAGGCCGGCGTGCCAGCTCCTATCGTGCCTACACACGGCGGCTTCGAAGTGACCACCA
GCGAGACAAGCACCCCCGCCATCCTGGATGTGTCTCAGGGCAGCAGCAACGTGCACATCAGCGTGAACACCTT
CAACAACCCCGCCTTCACCGAGCCCAGCGTGCTGCATCCTCCACCTCCAGTGGAAGCCAGCGGCAGACTTGTG
ATCAGCAGCTCCACCGTGTCCACCCAGAACTACGAAGAGATCCCCATGGACACCTTCGTGATCACCGGCGACC
ACCGGTTCAACACCACCAGCACACCTATCCCCGGCAGCAGACCTCCTGCCAGACTGGGCCTGTATGGCAGAGC
ACTGCAGCAAGTGCGGGTGGTGGATCCCGCCTTTCTGACCACACCCGCCAGACTGATCACCTACGACAACCCC
GTGTACGAGGGCGTGGACGATGCCACCCTGCAGTTCAGCCACCCCACCATCCACGAGCCCCCCGACCCTGACT
TCCTGGATATTGTGGCCCTGCACAGACCCGCCCTGACAAGCAGACGGGGCACAGTGCGGTTTAGCAGAGTGG
GCCAGAGGGCCAGCATGCACACAAGAAGCGGCGCCAGAATCGGAGCCCGGGTGCACTACTTTCAGGACCTGA
GCAGCATTGCCCCTGCCGAGGCCACAACCGAGAGCATCGAAATGCAGCCCCTGCTGCCTGCCGCCACACAGGA
CATCGACCTGTACGACATCTACGCCGTGGACGAGGACGTGACCTCTCCTGCCCAGCCTACCCTGCCTTTCCCAA
GCTCTACAGCCTCCGCCGTGGATGCCACACTGCCTTGGACATCCACCGTGCCTCTGAGCACCGGCCTGGACATC
ACACTGCAGCCTGGCCCCGATATCCCTCTGCAGTTTCCTCTGGCCGAGAGCCCCCTGCACCCTGTGACACCTCT
GACACCTATCGGCCACGTGGTGGTGCACGGCGGCGATTTCTATCTGCACCCCAGCTACTACACCCTGCACAAG
CGGCGGAAGCGGATGCCCAGATTTCTGGCCGATGTGTCCGTGGCCGCCTGA

MfPV11

L1

ATGAGCCTGTGGCGGCCTAGCGACGCCAAGGTGTACCTGCCTCCTGCCCCTGTGTCCAAGGCCATCAGCACCG
ACGAGTACGTGACCCGGACCAGCACCTACTACCACGCCGGCTCTAGCAGACTGCTGGCCGTGGGCCACCCTTA
CTACCCCGTGAAGAAGTCCAACGGCAAGATCGCCGTGCCCAAGGTGTCCGGCCTGCAGTACAGAGTGTTCAG
AGTGAAGCTGCCCGACCCCAACAAGTTCGGCCTGCCCGATGCCAACTTCTACGACCCCAATACCCAGCGGCTC
GTGTGGGCCTGCATCGGCATGGAAGTGGGCAGAGGACAGCCTCTGGGCGTGGGCACATCTGGACACCCCCTG
CTGAACAAGCTGGACGACACCGAGAACAGCCACAACAACGGCGCCAACCAGGGCACCGACAACCGCGAGTGT
GTGTCCATGGATTACAAGCAGACCCAGCTGTGCCTGCTGGGCTGCAAGCCTCCTACAGGCGAGCACTGGGGC
AAGGGCACCCCTTGTACATCTGGCGCCGATGGCGATTGCCCCCCTCTGGAACTGATCAACAGCGTGATCCAGG
ACGGCGACATGGTGGATGCCGGCTACGGCTGCCTGGATTTCCAGGCCCTGCAGACCAACAAGAGCGACGTGC
CCCTGGACATCTGCATGAGCACATGCAAGTACCCCGACTACCTGAAGATGGCCAGCGAGCCCTACGGCGACCG
GCTGTTCTTTTTCCTGCGGCGGGAACAGATGTTCGTGCGGCACATGTTCAACAGAGCCGGCACCATGGGCGAG
ACACTGCCCAGCGACCTGTACATCAAGGGCACAGGCAACAGAAGCAGCCTGGCCAGCCACATCTTCAGCAGCA
CACCCTCCGGCAGCATGGTCACCTCTGAGAGCCAGCTGTTTAACAAGCCCTACTGGCTGCAGCGGGCCCAGGG
ACACAACAATGGCATCTGCTGGGGCAACCAGGTGTTCCTGACCGTGGTGGACACCACCCGGTCCACCAATGTG
ACCCTGTGCGCCACCAAGACCAGCGAGGACACCTACAAGAACGACAACTTCCGCGAGTACCTGAGGCACATG
GAAGAGTTCGACCTGCAGTTCGTGTTTCAGCTGTGCAAGATCACCCTGACCACCGAAGTGATGGCCTACATCC
ACAACATGGACCCCAGCATCCTGGAAGATTGGAACTTCGGCGTGCAGCCCCCTCCTGCCGGAACACTGCAGGA
TACCTACAGATTCGTGCAGAGCGAGGCCATCCGGTGCCAGAAAACAGCCGCCCCTAAAGTGAAAGAGGACCC
CCTGAGCAAGTACACCTTCTGGGACGTGGACCTGCGGGACAAGTTCAGCGCCGACCTGGACCAGTTCCCCCTG
GGCAGAAAGTTCCTGCTGCAAGCTGGCATGCGGGCCAGAAGCACACTGAGAGCCCCCAAAAGACCCGCCCCT
ACCACAAGCAGCGGCAGCAGCAAGAAGAGAAAGACCACCAGAAGATGA

L2

ATGAAGCAGCCCGCCAGAGCCAGCAGAAGAAGGCGGAACGCCAACGGCCACGTGCACCGGCCCAGAAGAAA
AAGAGCCAGCGCCACCCAGCTGTACCAGACCTGTAAAGCCGCCGGAACCTGCCCCCCTGACGTGATCCCTAAG
GTGGAAGGCACCACCGTGGCCGACCAGATCCTGAGATACGGCAGCATGGGCGTGTACTTCGGCGGCCTGGGA
ATCGGAACAGCCCCTGGATCTGGCGGCAGATCTGGCTATGTGCCCCTGGGAAGCAGACCTGCCACAGTGCCTG
AGGTGCTGCCCAGACCTCCTGTGCTGGTGGAACCTGTGGCCCCCAGCGATCCTAGCATCGTGTCCCTGGTGGA
AGAGGCCAACCTGATCGATGCCGGACTGCCTGCCCCTAGCGTGCCAACAGGCGGAGGCTTTACCGTGACCACC
AGCGACGTGTCCACCCCCGCTATCCTGCCTGTGACACCAGCCGAGACAAGCGTGCACGTGACAGTGGACACCT
TCACCAACCCCCTGTTCACCGAGCCCAGCGTGTTCACCCCTCCACCCCCTATGGAAGCCACCGGCCACATCGTG
CTGAGCAGCGATACAGTGTCCGCCCACAGCTACGAAGAGATCCCCATGGATACCTTCGTCGTGACCGGCGACA
ACGCCTACAACCCTACCAGCACCCCCATCCCCACCCCCAGACCTAGAGCTAGACTGGGCCTGTACGGCAGAGG
CATGCAGCAAGTGCGGGTGTCCGATCCCGCCTTCCTGTCTAGCCCTGCCCGGCTGATCACCTTCGACAACCCTG
CCTATGAGGGCCTGCCCGAGGACAGCCTGCAGTTCGAGCACAGCAGCATCCACCAGCCCCCGACCCCGACTT
CCTGGATATTGTGGCCCTGCACAGACCCGCCCTGACCAGCAGACAGGGCACAGTGCGGTATAGCAGAGTGGG
CAACCGGGCCACCATCCGGACAAGAAGCGGCAAGCAGATCGGCGCCAGAGTGCACTTCTTCCAGGACATCAG
CGCCATCCCCCACCCTGAAGAGATCGAGATGCAGCCTCTGGTGTCTGCCCAGGAACCCCTGTTCGACGTGTAC
GCCGACCTGGAAGATGCCCCCGAAGTGGAAGGCGGCACAGGCAGCGCTACAAGCAGCTCTGTGCCTCCACTG
CAGGGCTCCGCCACCTGGAACACAACCGTGCCTCTGAACACCGGCCTGGACATCCTGGTGCAGCCTGGACCTG
ATGTGGCCCAGCAGTTTCCTGTGGCCGAGAGCCCTTACTGGCCCGCCATGCCAGTGTTTCCGCAGGGCCATGT
GTACGTGTCCGGCGGAGACTTTCTGTGGCACCCCTCCCTGTACACCCCTCGGCGGAAGAGAAAGCGGGTGCAC
ACCTTCTTCGCCGATGTGTCCGTGGCCGCCTGA

MmPV1

L1

ATGAGCATGTGGCGGCCCAGCGACAGCAAGGTGTACCTGCCTCCTGTGCCCGTGTCCAAGGTGGTGTCCACCG
ACGAGTACGTGTCCCGGACCAGCATCTACTACCACGCCGGCAGCTCTAGACTGCTGGCCGTGGGCCACCCTTA
CTACGCCGTGAAGAAAGGCAACAACAAGGTGTCCGTGCCCAAAGTGTCCGGCCTGCAGTACCGGGTGTTCAG
AGTGCGGCTGCCCGACCCCAACAAGTTCGGACTGCCCGATGCCAACTTCTACGACCCCAATACCCAGCGGCTC
GTGTGGGCCTGTCTGGGCGTGGAAGTGGGAAGAGGACAGCCTCTGGGAGTGGGCACATCTGGCCACCCCCTG
CTGAACAAGCTGGACGACACCGAGAACGGCCCCAAGCTGGCTGGCGGACAGGGCGCCGATAACAGAGAATG
CGTGTCCATGGATTACAAGCAGACCCAGCTGTGCATGCTGGGCTGCAAGCCTCCAGTGGGAGAGCACTGGGG
CAAGGGCAATCCTTGTACCACAGCCGCCGCTGGCGATTGCCCTGCTCTGGAACTCGTGAACAGCGTGATCCAG
GACGGCGACATGGTGGATACAGGCTACGGCGCCATGGACTTCAACGCCCTGCAGGCCAACAAGAGCGACGTG
CCCATCGACATCTGCACCAGCGTGTGCAAGTACCCCGACTACCTGAAGATGGCCAGCGACCCCTACGGCGACA
GCCTGTTCTTTTACCTGCGGCGCGAGCAGATGTTCGTGCGGCACCTGTTCAACAGAGCCGGCACCATGGGCGA
CAGCGTGCCCGACGATCTGTACATCAAGGGCAGCGGCAGCAACGTGAAGCTGGCCTCCCACGTGTTCTACCCC
ACACCCTCTGGCAGCATGGTCACCTCCGATGCCCAGCTGTTTAACAAGCCCTACTGGCTGCAGAAGGCCCAGG
GCCACAACAACGGCATCTGCTGGGGCAACCAGGTGTTCCTGACCGTGGTGGACACAACCCGGTCCACCAACAT
GACCCTGTGTGCCAGCACCGCCTCCACCGTGACCACCCCCTACAACAACGAGAGCTTCAAAGAATACCTGCGCC
ACGTGGAAGAGTTCGACCTGCAGTTCATCTTCCAGCTGTGTAAAGTGACCCTGAACACCGAAGTGATGGCCTA
CATCCACAGCATGGACGCCAGCATCCTGGAAGATTGGAACTTTGGACTGCAGCCACCCCCAAGCGGCAGCCTG
CAGGACACCTACAGATTCGTGACCAGCGCCGCCATCACCTGTCAGAAGCCTGCCCCCCCTAAAGAGAAAGAGG
ACCCCCTGGCCAAGTACACCTTCTGGGAGGTGGACCTGAAAGAGAAGTTCAGCGCCGACCTGGACCAGTTCCC
CCTGGGCAGAAAGTTCCTGCTGCAAGCTGGCATGCGGGCCAGACCTACACTGAGAGCCCCTAAGAGAACCGC
CAGCAGCACCAGCTCCAGCAGCCCCCGGAAGCGGAAACGGACCAAGAGATGA

L2

ATGAAGCATGCACACGTGTCGCGGCGCAAGCGAGCGGCCCCGCGCCCACCTGGTGGGCGGCAGAAGCGTGC
ATCTGCCACGCAGCTGTATCAAACCTGCAAGGCGGCAGGCACATGCCCCCCCGATGTTATCCCTAAGGTGGAA
GGCACAACCGTAGCAGATCAAATTTTAAAGTATGGCAGCATGGGTGTATATTTTGGGGGTTTGGGCATTGGCT
CTGGTGCTGGCACGGGCGGAAGAAGCGGCTACGTGCCCCTGGGTTCACGTCCCGCGTCGATTCCCGAGCCGTT
GCCACGACCACCGGTAACAATTGAGCCTGTAGGCCCTTCTGATCCATCCATTGTGTCATTGCTGGAAGAGTCCA
GACTAATAGAGGCAGGTGTACCAGCCCCAACATTCCCCACTCATGGGGGGTTTGAGATTAGCACATCTGAAGT
CAGCACACCCGCTATTCTGGATGTGTCTAGCAGTGGCTCTAATGTGCACGTCAGTGTAACCACCTTTACAAATC
CTACTTTTACTGAGCCATCTGTGTTGCGACCCCCGCCCCCTGTAGAGGCGTCTGGACGCCTGGTAATATCTGCA
TCATCTGTTAGCACGCATAGCTACGAGGAAATACCCATGGACACATTTGTAATAACTGGAGACCACAACTATAA
CACAACCAGCACACCCATTCCTGGTTCACGTGCCCCTGCACGACTTGGTCTATATGGACGTGCCACCCAGCAAG
TGCGGGTGGTGGATCCTGCATTTATAACTACCCCTGCGCGACTAGTGACATATGACAACCCTGCATATGAGGG
TGTGGACGATGCCACCCTGCAATTTTCCCATTCTGACATTCACCAGCCGCCAGATCCTGACTTCCTTGACATTGT
GGCATTGCACAGGCCCGCCTTGACCTCACGTAAGGGCACCGTGCGCTTTAGCCGATTAGGCCAGAGGGCAAC
ACTAACCACACGCAGTGGTAAGCGTATTGGGGCCAAGGTGCATTTCTATCATGACCTCAGTCCTATTGCCCCTG
CAGAAAGCATTGAGTTGCAGCCCCTGTCGTCTCAGGGAGAGCTGTATGACATATATGCAGATGTGGACGGGC
AAGAGGACGTTGCAGCTATGGCTGACACCCCATTAAACAGCAACAGCAGTGGTACTGCAAGCCCCTGGAACAC
CACAGTGCCCCTCAGTGCAGGGGCGGATGTGACTCTGCAGTCAGGCCCTGATGTGTCCCTGGATGCACCGGTG
GCTGAATCGCCTGTGCACCCTGGAGTGCCTTTAAGGCCTTCTGCACATATTATTCTGTATGGGGGAGACTTCTA
TTTGCACCCTAGCTACCTTGGTATTCGCAGGAAACGTAAACGCATGCACAATTTCTTTTCAGATGTCTATGTGGC
GGCCTAG

PcPV1

L1
ATGGCCGTGTGGCTGCCCGCCCAGAACAAGTTCTACCTGCCCCCTCAGCCCACCACCAGAGTGCTGCACACCG
ACGAGTACGTGACCCGGACCCGGATCTTCTACCACGCCAGCAGCGACAGACTGCTGACCGTGGGCCACCCATT
CTTCGACATCTACAAGAACCAGGAAATCATCGTGCCCAAGGTGTCCCCCAACCAGTACCGGGTGTTCCGGCTG
AGACTGCCCGACCCCAACAACTTCGCCTTCGGCGACAAGAGCCTGTTCAACCCCGAGAAAGAACGGCTCGTGT
GGGCCCTGAGAGGCCTGGAAATCGGCAGAGGACAGCCTCTGGGCGTGGGCGTGTCCGGCAACCCTACCTTCG
ACAGATACAGCGACGTGGAAAACGCCAACAAGAACCCCACCGGCCACGCCGACAATGCCCCCGACCCTAGAG
TGAATATGGCCGTGGACCCCAAGCAGACCCAGATGTTCATGGTGGGATGCAAGCCCGCCCTGGGCGAGCATT
GGGTCAAGGCCAGATGGTGCAATGGCGCCGCTCACGAGAGCCAGCAGTGCCCTCCTATCGAGCTGAAGAACA
CCCCCATCGAGGACGGCGACATGGTGGACATCGGCTTCGGCGCCATGGACTTCAAGAACCTGCAGCAGAACC
GCAGCGCCGTGCCCCTGGACATCATCGACACCCACTGCAAGTACCCCGACTACATCAAGATGGCCAACGACCC
CTACGGCGATACCTGCTTCTTCTTCGTGCGGCGGGAACAGCTGTACGCCAGACATCTGCTGGCCCGCTCTGGAC
AAGTGGGCGAGCCTGAGCCTGAGAGAACCGTGGCCACCAGAAGCACCTACCCCACCCTGAACTACTTCAGCA
GCCCCAGCGGCAGCCTGGTGTCTAGCGAAGCCCAGCTGTTCAACAGACCCTATTGGATCCAGCGGAGCCAGG
GCCAGAACAACGGAATCGCCTGGGAGAACCAGCTGTTTCTGACAGTGGCCGACAACACCAGAGGCACCCCCC
TGACCATCAACGTGGGCCCCAATGACAGAGCCGAGGATGGCGAGTACAAGGCCGGCAGCTACAAGACCTACC
TGCGGCACGTGGAAGAGTTCGACATCAGCGTGATCCTGCAGCTGTGCAAGGTGCAGCTGACCCCCGAGAATC
TGGCCACCATCCACACAATGAACCCCGATATCATCGAGAGCTGGCACCTGAACGTGAACCCCCCTTCTGGCGCC
CTGGACGACACCTACCGGTACATCACAAGCCTGGCCACAAAGTGCCCCACCAACGTGCCCCCCAAAGAGAGAG
AGGACCCTTACGCCCACCTGAAGTTCTGGGAGGTGGACCTGCGGGACAAGCTGACCGAGCAGCTGGATCAGA
CCCCCCTGGGCCGGAAGTTCCTGTTCCAGACCAATGTGCTGCAGGGCGGAGGCAGCAAGAGGGCCAGAGTGA
CCACAAGCGTGTCCAAGGACAAGCCCGTGAAGCGGCGGAGAGGCCAGAAATGA

L2
ATGCTGCTGAAGCGGCGGAAGAGAGCCGCCCCTAAGGACATCTACCCCCAGTGCAAGATCAGCAACACCTGTC
CCCCCGACGTGCTGAACAAGGCCGAGCAGTCTACCCTGGCCGACAAGATCCTGAAGTACGGCTCTGCCGGCGT
GTTCCTGGGCTCTCTGGGAATCGGAACAGGCAGAGGCTCTGGCGGCACACTGGGCTATGTGCCTGTGGGAAC
AGGACAGGGCGTGCGGCTGGGCACCAGAGTGTCTACAGTGCGGCCTAGCCTGCCCATCAGCTCTGTGGGCAC
AGCCGACGTGATCCCCATCGACGCTGTGGATCCTCTGGGACCTGCTGTGCTGCCCGGCAACGTGTTCCCTACCG
CCGTGGAAGATCCCTTCACCATCCAGCCCCCCAGATTCCCCAGCATCGTGGAAGAACCCGTGTCCGTGCACAGC
GAGAGCATCGTGACCGAGAGCGTGACAGAGGTGCCCGTGAACACCCCCAAAGTGACCATCGACGGACAGCCC
GCCGTGCTGGAAGTGGTGCCTGAGACAAGAGAGCCCCGGATCCTGAGCAGAAGCCAGTACGGCAACAGCGC
CTTCGAGGTGTCCCTGACAGCCTCTGCTGGCAGCGGCGAGACAAGCAGCAGCGACCACATTCTGGTGCACGG
CTTCACAGGCGGCCACGTGATCGGAGAGCAGATCCCCCTGCAGGAACTGGGCGGCAGATCCTTCAGCTCCACC
CTGGAAGCCGAGACAACCTTCACCACCTCCACCCCCAAGGCCGATGCCGTGGCCGAACCCAGAAGAGTGTTCA
CCAGCAGGCGGCTGGAACAGATCCCCGTGCGGGATCCTGGCTTCATCAGCAGACCCCGGTCCCTCGTGACCTT
CCAGAACCCCACCTTCGATGAGAGCGTGGACCTGTTCTTCGAGCGGGACGTGGCAGAACTGGCCCTGGCCGCT
CCCAACGAGGACTTCAGAGATCTGGTGTCTCTGAGCAAGCCCACCTTCAGCCGGACACTGGAAGGCAGAGTG
CGGGTGTCCAGACTGGGCACAAAGGCCACCATGAGAACCCGCAGCGGCCTCGTGATTGGCCCCCAGAGCCAC
TACTACTACGACCTGTCCGATATCGCCCCTGCCGAGAACCTGGAACTGACCCCCATCGGCAACATGAGCCTGG
GCGAACAGAGCGGACAGGCCGTGATCAGCAGCGGCACCAGCGACCTGGAAATCATCAGCCTGGAAAGCAGC
ACCATCGACAGCTACCCCGAAGAGTTCCTGCTGGCCGAGATCGAAAGCGTGGCCAACGACCTGCAGCTGGTGT
TCGGCGATAGAAGGGCCCAGCAGCCTATCAGCGTGCCCCACATTCAGAGGCCCAGCCCCCAGGTGTTCCCTCA
GTTTGAGGGCGTGTACGTGTCCCAGGGCACAGGCAGTGTGCCCCCTACCATCCCCACCGACCCTAACAAGACC
CCCGCCATCATTCTGGAAATCTGGGGCTCCGGCGAGAACTACAGCCTGCACCCAAGCCTGCTGAAGAGAAGAA
AGCGGAAGCGGCTGATTCTCTGA

PlPV1

L1

ATGGCCGTGTGGCTGCCCGCCCAGAACAGATTCTACCTGCCCCCTCAGCCCAGCACCAAGGTGCTGAACACCG
ACGACTACGTGACCCGGACCAGCATCTTCTACCACGCCGGCAGCGAGAGACTGCTGACAGTGGGCCACCCCCT
GTACGACATCTACGACGCCGAGAACGAGCACGTGATCGTGCCCAAGGTGTCCGCCAACCAGTACCGGGTGTTC
CGGATCAGACTGCCCGACCCCAACAACTTCGCCTTCGGCGACAAGGCCATCTTCGACCCTGAGAAAGAACGGC
TCGTGTGGGCCGTGCGGGGCCTGGAAATTGGAAGAGGACAGCCTCTGGGCGTGTGCGTGTCCGGCAACCCCC
TGTTCGATAAGAACAACGACGTGGAAAACCCCACCAAGTACTTCGCCAACCACGAGCAGGCCGACAACAGAG
TGAACGTGGCCTTCGACCCCAAGCAGACCCAGCTGTTCATGATCGGCTGCAAGCCCGCCATCGGCGAGCATTG
GGGACAGGCTAGAAGATGTGTGGGCGAGGGCCACACCCCAGGCCACTGTCCTCCAATCGAGCTGAAGAACAC
CACCATCGAGGACGGCGACATGATCGACATCGGCCTGGGCGCCATGGACTTCAGAGTGCTGCAGCAGAACAA
GGCCGGCGTGCCCCTGGACATCAGCAACAGCGAGTGCAAGTACCCCGACTACATCAAGATGGCCAACGACCC
CTACGGCGACAACCTGTTCTTCTACGTGCGGAGAGAGCAGCTGTACGCCCGGCACATGTTCACCAGAAGCGGC
AACCTGGGCAACGAGACAGTGCCCACCGATAGATACGTGAACCGGGCCGACAATACCATCCCCACCAGCAACT
ACTTCAGCACCCCCAGCGGCAGCCTGGTGTCTAGCGAGGCTCAGCTGTTTAACCGGCCCTATTGGATCCAGCG
GAGCCAGGGCCAGAACAACGGAATCGCCTGGCAGAATCAGCTGTTCATCACCGTGGTGGACAACACCCGGGG
CACCAGCCTGAACATCATCATGGGCAAGGACGACAAGACCGCCACAGGCGACTTCAACCCCGCCGACTACCGG
TGCTACATGCGGCACGTGGAAGAGTACGAGATCAGCCTGATCCTGCAGCTGTGCAAAGTGAAGCTGACCCCC
GAGAACCTGGCCTTCATCCACACCATGAACCCCGACATCATCGAGGATTGGCACCTGAACGTGAACCCCCCTGC
CGGCGCTATCGACGACGTGTACCGGTTCATCAACAGCCTGGCCACCAAGTGCCCCGACAACGTGCCCCCTAAG
ACCAGAGAGGACCCTTACGGCCTGTACAGATTCTGGGAGGTGGACCTGAAGGACAAGATGACCGAGCAGCTG
GACCAGACCCCCCTGGGCAGAAAGTTCCTGTTCCAGACCGGCGTGCTGCAGAGGCGGGCCAGACCTGCCAAT
AGAGTGTCCACCAGCACCACCAGACGGGCCGTGAAGAGAAAGCGGGCCAGCAAATGA

L2

ATGACCACCAGAAGCCGGAAGAGAAGGGCCGCTCCCAGAGACATCTACCCCAGCTGCAAGCTGGCCAACACC
TGTCCCCCCGACATCGTGGACAGCATCGAGAACAACACCCTGGCCGACAAGATCCTGAAGTACGGCTCTGCCG
GCGTGTTCTTCGGCTCTCTGGGCATCGGAACCGGCAGAGGCACAGGCGGCAGCACAGGCTATATCCCTCTGG
GAGAAGGCGCTGGCGTGCGGCTGAATACCAGAGTGTCTACCGTGCGGCCCAGCCTGCCTATCAGCAGCGTGC
ACCCTACCGACGTGATCCCCGTGGATGCCGTGGATCCTCTGGGACCTGCCATCGTGCCTCTGAGCGAGCTGCCT
AGCATCGTGGAAGATCCCGACCCCATCCTGCCCCCCAGATTTCCAACAGCCGTGGAAGAGAGCGTGATCGACT
TCAGCCCTGCTGGCCCTGGCGGCGATCTGCCTATCCAGAGCCCTAAAGTGACCACCACCGACACCAGCGCCCT
GATCGAAGTGACCCCCGAGACAAGACCCCCCAGAATCATCAGCAGAAGCCAGTACAGCAACCCCAGCTTCGA
GGTGCACATCACCAGCACCTCTGGCAGCGGCGAAAGCTCTGCCGTGGACCACGTGCTGATCGACGGCTACTCT
GGCGGAGAAGTGATCGGCGAGGAAATCCCTCTGATCGACCTGCAGAGCACCCGGTCCAGCAACACCTTCAGC
ACCACCGAAGTGCGGGAAACCAGCTTCTTCACCTCCACCCCCAGAGGGGAGCTGCCAAGCGCCAGACCTAGAA
CCCTGTACAACCGGCGGGTGCAGCAGGTGCAGGTGGTGGATCCTGCCTTCCTGAGCAGACCTGGCGCCCTCGT
GACCTTCGACAACCCCGCCTACACCGACGACGTGGAACTGATCTTCGAGCAGGACCTGGACGACCTGGCCAGA
GCCGCCCCTCACGAGGACTTCAGAGATATCGTGTCCCTGGGCAGACCCGTGTACGGCAGAAATCCTCAGGGCG
GCGTGCGGATCAGCAGACTGGGACAGAAAGCCACCATGCGGACCAGATCCGGCCTGAGAATCGGCCCCCAGA
GCCACTTCTTCTACGACATCAGCGAGATCGCCGAGCCCGAGCTGGAACTGGTGCCTCTGGAACCACCTTTCGTG
GGCGAGCAGACAGGCGAGAGCGTCGTGGGATCTGCCCTGGGCGAGTTCGAGACAATCAGCCTGAGCAACGA
GCCCGCCATCTACCCTGAGGACACCCTGATTGACGAGTACGAGGTCGTGGGCAGCGATCTGCAGCTGATCATC
GGCGATAGCGGAGGCGAGAGGCCTATCCCTGTGGCCGACTTTGCCAGACCCCCTGCCAAGCTGTTCCCTGAGC
TGGATGGCGTGCAAGTGATCAACGGCCGGGACGTGTCCAGATCCGCCACCGTGCCTGTGATTCCCGAGGATA
CCCCCCTGATCATCATCGAAGTGCTGGACGGCTCCGGCGACTACTTCCTGCACCCCAGCCTGTTCCGGAAGCGG
CGGAAGAGGCCATTCTTCTGA

<u>PtPV1</u>

L1

ATGTGGCGGCCCAGCGACAACAAGCTGTACGTGCCACCTCCTGCCCCCGTGTCCAAGGTGCTGACCACAGATG
CCTACGTGACCCGGACCAAGATCTTCTACCACGCCAGCAGCAGCAGACTGCTGGCCGTGGGCAACCCCTACTT
CCCCATCCGGAAGGCCAACAAGACCATCGTGCCCAAGGTGTCCGGCTTCCAGTTCCGGGTGTTCAAGATCGTG
CTGCCCGACCCCAACAAGTTCGCCCTGCCCGACACCAGCATCTTCGACAGCACCAGCCAGAGACTTGTGTGGG
CCTGCATCGGCCTGGAAGTGGGCAGAGGACAGCCTCTGGGCGTGGGCTATTGTGGCCACCCCTGCCTGAACA
AGTTTGACGACGTGGAAAACAGCGCCAGCTACGCCGTGAACCCCGGCCAGGACAACAGAGTGAACGTGGCCA
TGGACTACAAGCAGACCCAGCTGTGCCTCGTGGGCTGTGCTCCTCCACTGGGAGAGCACTGGGGCAAGGGCA
AGCAGTGTTCTGGCGTGTCCGTGCAGGACGGCGATTGCCCTCCTCTGGAACTCGTGACCAGCGTGATCCAGGA
TGGCGATATGGTGGACACCGGCTTCGGCGCCATGGATTTCGCCGAGCTGCAGAGCAACAAGAGCGACGTGCC
CCTGGACATCTGCACCAGCACATGCAAGTACCCCGACTACCTGCAGATGGCCGCCGACCCCTATGGCGACCGG
CTGTTCTTCTACCTGCGGAAAGAACAGATGTTCGCCCGGCACTTCTTCAACAGAGCCGGCACAGTGGGCGAGC
AGATCCCCGATGAGCTGTTCGTGAAGGGCACCACCAGCAGAGCCACCGTGTCCAGCAACATCTACTTCAACAC
CCCCAGCGGCAGCCTGGTGTCTAGCGAGGCCCAGCTGTTCAACAAGCCCTACTGGCTGCACAAGGCCCAGGG
CCACAACAACGGCATCTGCTGGGGCAACACCCTGTTTGTGACCGTGGTGGATACCACCCGCAGCACCAACATG
ACCGTGTGCGCCAGCACCACCTCTAGCCCTAGCGCCACATACACCGCCAGCGAGTACAAGCAGTACATGCGGC
ACGTGGAAGAGTTCGACCTGCAGTTCATCTTCCAGCTGTGTACCATCAAGCTGACAGCCGAGCTGATGGCCTA
CATCCACACCATGAACCCCACCGTGCTGGAAGAGTGGAACTTCGGCCTGAGCCCCCCTCCCAATGGCACCCTG
GAAGATACCTACAGATACGTGCAGAGCCAGGCCATCACCTGTCAGAAGCCCACCCCCGACAAAGAGAAGCAG
GACCCTTACGCCGGCCTGTCCTTCTGGGAAGTGAACCTGAAAGAGAAGTTCAGCAGCGAGCTGGAACAGTAC
CCCCTGGGCCGGAAGTTCCTGCTGCAGACAGGCGTGCAGTCTACCAGCCTGGCCAGAGCCGGAACAAAGAGA
GCCGCCAGCACAAGCACCGCCACCCCCACCAGAAAGAAAGTGAAGCGGAAATGA

L2

ATGGCCCACAGCCGGCCCAGAAGAAGAAAGAGAGCCAGCGCCACCCAGCTGTACCAGACCTGCAAGGCCAGC
GGCACCTGTCCCGACATCATCCCCAAGGTGGAACAGAACACCCTGGCCGACAAGATCCTGAAGTGGGGCAGC
CTGGGCGTGTTCTTTGGCGGCCTGGGAATCGGCACAGGCTCTGGCACAGGCGGCAGAACAGGCTACGTGCCA
CTGGAAAGCGCCCCCAGACCCGCCATCCCTTTTGGCCCAACAGCCAGACCCCCTATCGTGGTGGATACCGTGG
GCCCCACCGACAGCAGCATCGTGTCCCTGGTGGAAGATAGCGCCATCATCAACAGCGGCGCCAGCGACCTGG
TGCCTTCTATCCACGGCGGCTTCGAGATCAGCACCAGCGAGAGCACAACCCCTGCCATCCTGGACGTGTCCATC
ACCACCCACAACACCACCAGCACCTCCATCTTCCGGAACCCCGCCTTCGCCGAGCCTAGCATCGTGCAGTCTCA
GCCCTCTGTGGAAGCCGGCGGACATCTGCTGACCTCCACCTTCACCTCCACCATCAGCCCCCACAGCGTGGAAG
AGATCCCCCTGGACACCTTTATCGTGTCCAGCAGCAACAGCAACCCCGCCAGCAGCACCCCTGTGCCTACAACA
GTGGCCAGACCCAGACTGGGCCTGTACAGCAAAGCCCTGCATCAGGTGCAAGTGACCGACCCTGCCTTCCTGA
GCAGCCCCCAGAGACTGATCACCTTCGACAACCCCGTGTACGAGGGCGAGGACATCAGCCTGCACTTCGAGCA
CAACAGCATCCACGAGCCCCCCAACGAGGCCTTCATGGACATCATCAGACTGCACAGGCCCGCCATCACCAGC
AGACGGGGAGTTGTGCGGTTCAGCAGAATCGGCCAGCGGGGCAGCATGTACACCAGAAGCGGCAAGCACAT
CGGCGGCAGAGTGCACTTCTTCACCGATATCAGCCCCATCAGCGCCGACGCCCAGGACATTGAACTGCAGCCT
CTGGTGGCCGCTGCCCAGGACGATAGCGACCTGTTCGACATCTACGTGGACCCCGACACCACCCCCGTGGCCG
TGGACAATATCCCCAGCGCCAACTCCACCCTGTTCATCAAGAGCAGCATCTTCGACACCAGCTGGGGCAATACC
ACCATCCCCCTGAGCCTGCCCAACAACATCTTCGTGCAGCCTGGCCCCGACATCCTGTTCCCTACAACACCAGCC
GTGCCCCCCTACGGCCCTGTGATTTCTCCTCTGCCTGTGGGCCCCGTGTTCATCAGCGGCAGCGAGTTCTACCT
GCACCCCTCCCTGTACTTCGCCCGGAAGAGGCGGAAGCGGGTGTCCCTGTTCTTTAGCGACGTGGCCGCCTGA

RaPV1

L1
ATGACCCAGCTGCACATGACCACCGGCCTGATCTACACCTGTACCTGCCTGGGCAAGAGCGCCAATGGCGTGC
CATCTGCCCTGCTGCAGATGGCCCTGTGGATCCCATCTCCCCAGGCCGTGTATGTGGCCCCTGCCCCTGTGACC
ACCATCCCCAGCACCGAGGACTTCATCACCCGGACCCCCTACTTCTACCACGCCAACAGCGACCGGCTGCTGAC
CGTGGGCAATCCCTTCTACGCCATCAAGGACCCCGGCACCCAGAAAATCCTGGTGCCCAAGGTGTCCGGCAAC
CAGTACCGGGTGTTCCGGATCAGATTCCCCGACCCCAACAAGTTCGCCCTGCCCGACCCTAACGTGTTCAACCC
CGACACCGAGAGACTTGTGTGGGGCCTGAGAGGCATCGAAGTGGGCAGAGGCGGACCCCTGGGCATGGAAG
TGACAGGCAATCTGGGCTTCGGCCGGAACGCCGACGTGGAAAACCCTAACCAGGCCGAGAGGGAACATGGC
GCCGTGGGCACCAAGAGATTCAACGTGGGAATGGAACCCAAGCAGAACCAGCTGCTGATTGTGGGCTGTAGC
CCAGCCTGGGGCGAGTTCTGGGATAGCACCCCTGCTTGCACCGAGGGCGATACCCCAGTGGATCCTGGCGCT
GGCGATTGCCCTGCCCTGGAACTGAAGTCCACCAGACTGCAGGACGGCACCATGACAGACATCGGCTTTGGCC
ACATGAACTTCAAGAGCCTGCAGGATGACAAGAGCGGCGTGCCCCTGGAAATCGTGAACAGCGTGTGCGTGT
ACCCCGACTTCTACAAGATGAGCAAGGACCCTTACGGCAACAGCTGCTTCTTCAGCGTGCGGAAAGAACAGAT
GTATATCCGGCACTACTTCAGCAGAGTGGGCGCCTACGGCGACACCGTGCCCACCGATATGTACCTGAAGGAC
AAGACCAACGGCGGAGCCAACTGGACCGGCCCTGTGTACATGGGAACACCCAGCGGCAGCATCGTGTCTACC
GAGGGCCAGGTGCTGAACCGGCCTTACTGGCTGCTGAAGGCCCAGGGCAGAAACAACGGCATGCTGTGGGG
CAATCAGTGCTTCGTGACCGTGGTGGACAATACCAGAAGCCTGAACTTCCTGATCAACGTGAAGAACGACGCC
GGCACCAGCTTCCAGGCCGACGAGTTCGCCAACTACCTGCGGCACACCGAGGAATACGAGATCGCCTGCATCG
TGCAGCTGTGCAAAGTGCGGCTGGACCCCGAGACACTGAGCATCCTGAACACCATGGACCCTGAGATCCTGG
AAGAGTGGCAGATCGGCGTGAACCCCCCTGTGTCCAGCCAAGTGAACGACCGGTACAGATTCGTGCACAGCC
TGGCCACCCACTGCCCCGACAAAGAGAAGGCCAAAGAGAAAGAGGACCCCTACGCCGGCCTGGCCTTCTGGA
ACCTGGATTTCACCGAGAGCCTGAGCCCCGACCTGGACCAGTTTCCTCTGGGCAGACGGTTCCTGACACAGGC
CGGCAGAGCCGGAAGAACCAGCGGCACAAGAAGCACCGCCAGAACCGGCAGAACAGGCACCGTCGTGAAGC
GGTCCATCGTGTCCACAACCGTGGCCGGCAAGCCTAGAAGCGTGCCCGCTAAGCGGCGGAGAAGATGA

L2
ATGGCCCCCAGAAAGCGGAGAGCCACCAGAGTGCCTAGAGACAGCGCCACCAACCTGTACAGACAGCCCGGC
TGCAGAGTGGACGGCAACTGTCCTTGGGGCGTGAAAGAGCAGATCGAGAACAAGACCCCTGCCGACCGGATC
CTGCAGTACGGCAGCGCCGTGATCAATCTGGGCGGCCTGGGAATCGGAACAGGCGCTGGATCTGGCGGCAG
AGGCGGCTATATTCCCATGGGCGCCGATAGAGGCATCGGCGTGGGAGCTTGGCCTAGACCTGCCTACCCTGCC
AGACCTGTGGTGCCCGCCATTGAGACAGTGGGCCCCACCATCAGCATCCCCGAAGTGGTGGCCACAGACGTG
ATCGAGATGGAACCCATCGTGACCGCCGTGGACCCCAGCGTGATCGATACCCACCCCCCCATCGACCCTACCG
ACCCTGCCATCGTGGAAGATTTCGGCAGCTACCCCCCCAGACCCGCCATCATTGATGAGAGCGTGCCAACCCA
GGGCGGCAACAGAATTCAGGTGGTGGCCGAGGTGCACCACCCCGCCGATCTGTTTCCAAGCACCACCATCTCT
AGCGGCGGCTCCACAACAAGCGCCGTGCTGGAAGTGGGCGAGCAGATCCCCCTGATGCCCAGAAGCAACCCC
CCCCACATCCACGAGCCTAGCCTGCTGACCACCACAACCAGCTTCGGCACCAACGCCGACGTCGTGGGAAGCA
GAGCCAGCATCATCGACTTCGACGCCGTGGATGAAGCCGTGGGCGACGACATCCCTCTGCTGGACAGAACCTA
CGACCGGAACGCCAACCTGGAATTCCGGACCAGCACACCCCAGAGCGGCAGACGGCCTAATCCTGTGAAGGC
CCTGAAGTCCCTGTACAACAAATACGTGCGCCAGGTGCCAGTGGAAGATCCCCTGTTCATGGAAGCCCCTGGC
CACCTGATCGAGTTCGGCAACCCTGTGTTCCAGCCCGAGGAAAGCCTGGAATACCCACTGCAGGACAACCCCC
TGGCCTCCCCCGATGAGAGACTGCAGGGAACCCACAGACTGCACCGGCCCATCCTGTCTGAAGTGCCAGGCG
GCAGGGGCATCAGACTGTCTAGACTGGGAGCCATCGGCGCCATGCGGATGAGATCTGGCCTGACCGTGGGCC
CTAGAGTGCACGTGTACCACGACATCAGCAGCATCGAGGAAGCCATCGAGCTGCAGCCCCTGGGCGTGGAAC
CTCACTCTGTGACAGGCGAGGCCGTGATGCAGGACACCTCTGTGGATGCCCTGACCGAAGAGGACATCACCG
AGCAGGGCTTCGAGGACGTGCCACTGCTGTCTCCACATGCCGGACAGCAAGTGCGCCTGCAAGTGGGACCTG
GCGGACGGAACAACCGGAACGTGGTGTCCCTGGAAATCCCCACCACCAACCGGGCCGACACCTTCGGCATCA
ATATCGGCGAGGCCAGCGACATCTACGTGCACTACGCCGACGAGAAGCACACCATCCCCGGCTTCGTGCCTGG

CATTCCTCTGGGACCTGCTGTGCCCCCTGTGATTGTGGAAGATGACACCGCCGCCTACGACTATTGGTTCGACC
TGTACCTGCATCTGCCCCGGAAGAAACGGAAGTGGTGCAGCTTCTGCAGCCTGACCGACGGCATCGTGGACAC
CTGA
